# DIRECTEUR: transcriptome-based prediction of small molecules that replace transcription factors for direct cell conversion

**DOI:** 10.1093/bioinformatics/btae048

**Published:** 2024-01-25

**Authors:** Momoko Hamano, Toru Nakamura, Ryoku Ito, Yuki Shimada, Michio Iwata, Jun-ichi Takeshita, Ryohei Eguchi, Yoshihiro Yamanishi

**Affiliations:** Department of Bioscience and Bioinformatics, Faculty of Computer Science and Systems Engineering, Kyushu Institute of Technology, Iizuka, Fukuoka 820-8502, Japan; Department of Bioscience and Bioinformatics, Faculty of Computer Science and Systems Engineering, Kyushu Institute of Technology, Iizuka, Fukuoka 820-8502, Japan; Department of Bioscience and Bioinformatics, Faculty of Computer Science and Systems Engineering, Kyushu Institute of Technology, Iizuka, Fukuoka 820-8502, Japan; Department of Bioscience and Bioinformatics, Faculty of Computer Science and Systems Engineering, Kyushu Institute of Technology, Iizuka, Fukuoka 820-8502, Japan; Department of Bioscience and Bioinformatics, Faculty of Computer Science and Systems Engineering, Kyushu Institute of Technology, Iizuka, Fukuoka 820-8502, Japan; Research Institute of Science for Safety and Sustainability, National Institute of Advanced Industrial Science and Technology (AIST), Tsukuba, Ibaraki 305-8569, Japan; Department of Bioscience and Bioinformatics, Faculty of Computer Science and Systems Engineering, Kyushu Institute of Technology, Iizuka, Fukuoka 820-8502, Japan; Department of Bioscience and Bioinformatics, Faculty of Computer Science and Systems Engineering, Kyushu Institute of Technology, Iizuka, Fukuoka 820-8502, Japan; Department of Complex Systems Science, Graduate School of Informatics, Nagoya University, Nagoya, Aichi 464-8601, Japan

## Abstract

**Motivation:**

Direct reprogramming (DR) is a process that directly converts somatic cells to target cells. Although DR via small molecules is safer than using transcription factors (TFs) in terms of avoidance of tumorigenic risk, the determination of DR-inducing small molecules is challenging.

**Results:**

Here we present a novel in silico method, DIRECTEUR, to predict small molecules that replace TFs for DR. We extracted DR-characteristic genes using transcriptome profiles of cells in which DR was induced by TFs, and performed a variant of simulated annealing to explore small molecule combinations with similar gene expression patterns with DR-inducing TFs. We applied DIRECTEUR to predicting combinations of small molecules that convert fibroblasts into neurons or cardiomyocytes, and were able to reproduce experimentally verified and functionally related molecules inducing the corresponding conversions. The proposed method is expected to be useful for practical applications in regenerative medicine.

**Availability and implementation:**

The code and data are available at the following link: https://github.com/HamanoLaboratory/DIRECTEUR.git.

## 1 Introduction

Cell therapy, the transplantation of normal cells to a damaged organ, is a promising approach for regenerative medicine. Induced pluripotent stem cells (iPSCs) are generally used for establishing target cells (i.e. healthy cells required for treatment) from somatic cells. Direct reprogramming (DR) is an alternative technique for converting somatic cells (source cells) to target cells via a single-step conversion without the use of iPSCs (Xu *et al.* 2015, Volarevic *et al.* 2018, Wang *et al.* 2021). Because cells converted by DR are less damaged by genomic mutations, the cancerization risk after cell transplantation is expected to be significantly reduced (Davis *et al.* 1987, Ferber *et al.* 2000). Thus, DR-based cell generation is suitable for the treatment of damaged tissues (Grath and Dai 2019). A previous study reported that mouse embryonic fibroblasts (MEFs) can be converted to myoblasts by overexpressing the transcription factor (TF) MyoD (Davis *et al.* 1987), and several methods of TF-based DR induction have since been developed using experimental (Ieda *et al.* 2010, Caiazzo *et al.* 2011, Sekiya and Suzuki 2011) and computational approaches (Cahan *et al.* 2014, D’Alessio *et al.* 2015, Rackham *et al.* 2016, Jung *et al.* 2021). Nevertheless, because of the use of viral vectors to overexpress TF-coded genes, a risk of tumorigenesis caused by viral insertional mutagenesis remains for TF-induced DR (Isomi *et al.* 2021).

To reduce the risk of tumorigenesis caused by gene insertions, induction of DR by small molecules such as drugs or chemical agents has been proposed (Federation *et al.* 2014, Qin *et al.* 2017, Xie *et al.* 2017). Small molecules have the potential to induce DR by regulating (i.e. activating or inhibiting) multiple signaling pathways, modifying epigenetic patterns (e.g. histone acetylation and methylation), and manipulating the expression level of TF-coded genes (Han *et al.* 2017). Small molecule combinations have been successfully used to convert somatic cells into cardiomyocytes (Fu *et al.* 2015) and neurons (Li *et al.* 2015). Furthermore, DR induced by small molecules enables *in vivo* reprogramming without cell transplantation. Notably, *in vitro* and *in vivo* experiments have allowed the conversion of cardiac fibroblasts into cardiomyocytes in the heart (Mohamed *et al.* 2017, Sadahiro and Ieda 2021), and *in vivo* experiment on mouse brain successfully converted astrocytes into neurons *in situ* (Ma *et al.* 2021, Guo *et al.* 2014). However, as more studies have focused on TF-induced DR, the small molecule combinations required for inducing DR in specific tissues is still poorly known. Thus, the development of a novel method for *in silico* identification of small molecules combination for DR induction is a challenging issue.

Because of cost and expense, identifying the optimal combination of small molecules for DR is difficult using *in vitro* experiments alone. There is a previous work on the optimization of small molecule combinations that encompass the biological pathways identified from proteins targeted by known DR-inducing small molecules (Nakamura *et al.* 2022). However, the previous method cannot be applied to cell conversions for which DR-inducing small molecules have not been experimentally identified yet. A regression-based method with the biological pathway profiles was proposed, but the gene expression alteration during the DR induction was not taken into account (Napolitano *et al.* 2021).

In this study, we developed a novel computational method, DIRECTEUR (DIrect REprogramming by Chemical with TranscriptomE Utilization for Regenerative medicine), to identify new combinations of small molecules for DR from approved drugs by integrating heterogeneous transcriptome data. Using large-scale small molecule-induced transcriptome profiles and TF-induced transcriptome profiles acquired from TF-based DR experiments, we performed a variant of simulated annealing to explore small molecule combinations with similar gene expression patterns with DR-inducing TFs. We applied DIRECTEUR to predicting combinations of small molecules that convert fibroblasts into neurons or cardiomyocytes, and were able to reproduce experimentally verified and functionally related molecules inducing the corresponding conversions. The proposed method is expected to be useful for practical applications in regenerative medicine.

## 2 Materials and methods

### 2.1 Construction of target cell-specific transcriptome profiles

Transcriptome profiles of target cells induced by DR through the overexpression of TFs in mouse fibroblasts were acquired. More precisely, we focused on the DR of mouse embryonic fibroblasts into neurons and cardiomyocytes. Transcriptome profiles of fibroblasts (source cells) and neurons (target cells) were acquired from GSE22292 (Caiazzo *et al.* 2011) in the Gene Expression Omnibus Database (GEO) (Barrett *et al.* 2007). Similarly, transcriptome profiles of fibroblasts (source cells) and cardiomyocytes (target cells) were acquired from GSE27174 (Ieda *et al.* 2010). Gene expression values of the source cells and target cells were compared, and a transcriptome profile z comprising log_2_ ratios was constructed. The number of genes was 25023 in the neuron-specific transcriptome profile and 24947 in the cardiomyocyte-specific transcriptome profile.

### 2.2 Construction of TF-induced transcriptome profiles

Transcriptome profiles in response to gene overexpression of TFs for DR were acquired. Ascl1, Lmx1a, and Nurr1 are TFs that induce neurons from fibroblasts (Caiazzo *et al.* 2011), and Tbx3, Mef2c, and Gata4 are TFs that induce cardiomyocytes from fibroblasts (Ieda *et al.* 2010). Thus, transcriptome profiles in response to gene overexpression of Lmx1a, Nurr1, Tbx3, and Mef2c were acquired from the L1000 mRNA profiling assay in the LINCS project (Subramanian *et al.* 2017) (https://lincsproject.org). Transcriptome profiles of the L1000 mRNA profiling assay were acquired from GEO (GSE70138 and GSE92742). The dataset consists of a total of 591855 transcriptome profiles, in which gene expression values were measured using 93 human cell lines with various perturbations. When there were multiple transcriptome profiles of the same gene, they were averaged. A transcriptome profile with each TF-coding gene overexpression was defined as $x^{\mathrm{oe}}={\left(x_{1},x_{2},\;\ldots ,\;x_{p}\right)}^{T},$ where *p* is the number of landmark genes (*p *=* *978).

We constructed a TF-induced transcriptome profile for each cell type by an average of transcriptome profiles with TF-coding gene overexpression, which was defined as $x^{\mathrm{TF}}$. In the case of neurons, transcriptome profiles with gene overexpression of Lmx1a and Nurr1 were averaged to construct ${x_{\mathrm{neuron}}}^{\mathrm{TF}}$. In the case of cardiomyocytes, transcriptome profiles of Tbx3 and Mef2c were averaged to construct ${x_{\mathrm{cardio}}}^{\mathrm{TF}}$.

### 2.3 Construction of small molecule-induced transcriptome profiles

The number of small molecules was 21182. We obtained 312596 small molecule-treatment profiles (denoted as “trt_cp”) from the L1000 mRNA profiling assay. For each small molecule, the corresponding International Chemical Identifier code (InChIKey) was also obtained from GEO. For each small molecule, we represented a small molecule-induced transcriptome profile by a feature vector defined as $y={\left(y_{1},y_{2},\;\ldots ,\;y_{p}\right)}^{T}$, where *p* is the number of genes (*p *=* *978). Of these, 1486 small molecules were registered in KEGG (Kanehisa *et al.* 2009), and 36 small molecules were experimentally confirmed to induce DR (Sizykh *et al.* 2021).

### 2.4 DR-characteristic transcriptome signatures

To identify DR-characteristic gene expression signatures, we extracted common patterns between target cell-specific transcriptome profiles and TF-induced transcriptome profiles by calculating the correlation coefficients between them. Since not all genes are involved in DR, we removed genes that did not contribute to the common gene expression patterns and selected genes that constituted the DR-characteristic gene expression patterns as DR-characteristic genes.

To calculate the correlation coefficient for organism-different transcriptome profiles, genes were converted to gene orthologs in the target cell-specific transcriptome profile z (derived from mice) and the TF-induced transcriptome profile x (derived from humans). Here, the target cell-specific transcriptome profiles consisting of common gene orthologs were defined as $z^{\prime }={\left({z^{\prime }}_{1},{z^{\prime }}_{2},\;\ldots ,\;{z^{\prime }}_{r}\right)}^{T}$ and the TF-induced transcriptome profile was defined as $x^{\prime }={\left({x^{\prime }}_{1},{x^{\prime }}_{2},\;\ldots ,\;{x^{\prime }}_{r}\right)}^{T}$, where r was the number of common orthologs (r = 594).

To identify commonly altered genes across the target cell-specific transcriptome profile and TF-induced transcriptome profiles, correlation coefficients between $x^{\prime }$and $z^{\prime }$ were calculated to select highly correlated orthologs. The threshold values of the correlation coefficient were set at 0.5, 0.55, 0.6, 0.65, 0.7, 0.75, 0.8, 0.85, 0.9, 0.95, and 1.0. In the case of the correlation coefficient of 1, the number of orthologs was less than the number of the overexpressed TFs (=3); hence, DR-characteristic gene expression patterns in neurons and cardiomyocytes were not identified at the threshold of 1. We constructed the target cell-specific transcriptome profile $z^{\prime }$ that consisted of common orthologs based on the correlation coefficient analysis, defined it as ${z\prime }_{\mathrm{DR}}$= ${\left({z^{\prime }}_{1},{z^{\prime }}_{2},\;\ldots ,\;{z^{\prime }}_{s}\right)}^{T}$, and referred to it as DR-characteristic transcriptome signature. Here, *s* is the number of correlated orthologs selected on the basis of correlation coefficient analysis. We also constructed small molecule-induced transcriptome signature $y^{\prime }={\left({y^{\prime }}_{1},{y^{\prime }}_{2},\;\ldots ,\;{y^{\prime }}_{s}\right)}^{T}$ that consisted of only correlated orthologs on the basis of correlation coefficient analysis from small molecule-induced transcriptome profiles.

### 2.5 Transcriptome-based prediction of the combination of DR-inducing small molecules by simulated annealing (DIRECTEUR)

We formulate a combinatorial optimization problem to identify the optimal combination of small molecules for DR, and propose a novel combinatorial optimization algorithm for solving it on the basis of simulated annealing in order to identify the optimal combination of small molecules. We attempted to predict a new combination of small compounds that induce DR from a candidate set of 1486 approved drugs and 36 known DR-inducing small molecules.

To explore new DR-inducing small molecules from the candidate set of *n* (*n *=* *1522) small molecules, the $I_{k}=\{i_{1,}\ldots ,i_{k}\}\;\left(1\leq k\leq n\right)$ index, a vector consisting of the selected small molecules $c\left(I_{k}\right):={\left({c\left(I_{k}\right)}_{i}\right)}_{i=1,\ldots ,n}\in {\left\{0,1\right\}}^{n}$ with an *n*-dimensional binary vector, was defined as follows:

$${c\left(I_{k}\right)}_{i}=\left\{\begin{array}{cc} 1, & if\;i\in I_{k}\\ 0, & \;\mathrm{otherwise} \end{array}\right.,$$

where the selected small molecules were set to 1 and the unselected small molecules were set to 0.

Considering the experimental cost and possibility for clinical application, it is ideal to have a limited number of small molecules. To restrict the number of small molecules to be selected, the subobjective function $f_{\mathrm{mole}}$ was defined as follows:

fmoleIk= 1                          if ∑i∈IcIki≤T   exp -∑i∈IcIkit2 otherwise, 

where *t* is the number of TFs that induce DR and *T* is the maximum number of small molecules that should be included in each combination (*T* was set to 10 in this study). As the number of selected small molecules increases, the value of $f_{\mathrm{mole}}$ decreases exponentially.

Next, to evaluate the similarity between the small molecule-induced transcriptome patterns and DR-characteristic transcriptome patterns, the subobjective function $f_{\mathrm{corr}}$ was defined as follows:

$$f_{\text{corr}}\left(I_{k}\right)=\mathrm{corr}\left({\bar{y}}_{I_{k}},\;z_{\mathrm{DR}}^{\prime }\right),$$

where ${\bar{y}}_{I_{k}}$ is the sum of the small molecule-induced transcriptome signatures $y^{\prime }$ with the specified indeces.

Finally, to identify the optimal combination of small molecules for DR, we defined a combinational optimization problem as follows:

$$\left|\begin{array}{l} \text{maximize}\;{f\left(I_{k}\right)=f}_{\text{corr}}\left(I_{k}\right)+f_{\text{mole}}\left(I_{k}\right),\\ \text{subject}\;\text{to}\;I_{k}\subset \{1,\ldots ,n\}, \end{array}\right.$$

where $f\left(I_{k}\right)$ is an objective function that has a value from 0 to 2.

$$f\left(I_{k}\right)\in \left[0,2\right]$$

In this study, simulated annealing (Kirkpatrick *et al.* 1983, Černý 1985), which is one of the local search methods in meta-heuristics, was applied to acquire the solution to the combinatorial optimization problem. We propose the following algorithm:

**Step 1:** Arbitrarily choose k satisfying 1≤k≤n and randomly select a set $I_{k}$ of *k* small molecules as the initial state. In other words, generate an *n*-dimensional 0–1 vector $c(I_{k})$ with *k* elements of 1 and (*n* − *k*) elements of 0. Then, calculate the objective function $f\left(I_{k}\right)$.

**Step 2:** Randomly select whether to increase the number of small molecules by one to generate a neighborhood solution or to decrease the number of small molecules by one to generate a neighborhood solution.

1) In the case of increasing by one:
  Randomly select one of the small molecules that have not yet been selected and add it to the candidate combinations. In other words, randomly select one of the elements of $c\left(I_{k}\right)$ that is 0 and change it to 1, and set the *n*-dimensional 0–1 vector as the neighborhood solution $c\left(I_{k}^{*}\right)$.
2) In the case of decreasing by one:
  Randomly select one of the small molecules from the already selected small molecules and remove it from the candidate combination. In other words, randomly select one of the elements of $c\left(I_{k}\right)$ that is 1 and change it to 0 and set the *n*-dimensional 0–1 vector as the neighborhood solution $c\left(I_{k}^{*}\right)$.Then, the objective function $f\left(I_{k}^{*}\right)$ is calculated.

**Step 3:** If $f\left(I_{k}^{*}\right)>f\left(I_{k}\right)$, set $I_{k}≔I_{k}^{*}$ with probability 1. Otherwise, $I_{k}≔I_{k}^{*}$ with the probability exp(−($f^{*}$−f)⋅iteration/n).

**Step 4:** If a predetermined fixed time has elapsed, let $I_{k}$ be the final solution. Otherwise, return to Step 2.

Here, the number of iterations was within 1000000. We defined the highest objective function score as the prediction score. Note that in the original annealing method, the end temperature was set as the end condition of the algorithm, but the calculation time was set as the end condition of the algorithm in this study.

### 2.6 Evaluation of biological functions of small molecules target protein groups

To acquire the list of target proteins regulated by predicted small molecules for DR, we collected a set of interactions between small molecules and proteins. These interaction data were acquired from public databases such as ChEMBL (Gaulton *et al.* 2012), MATADOR (Günther *et al.* 2008), DrugBank (Knox *et al.* 2011), BindingDB (Liu *et al.* 2007), KEGG DRUG (Kanehisa *et al.* 2009), Psychoactive Drug Screening Program Ki (PDSP-Ki) (Roth *et al.* 2000), and Therapeutic Target Database (Qin *et al.* 2014). This dataset included 1522 small molecules and 2470 proteins, along with 18650 interactions.

GO and KEGG pathway enrichment analyses were performed to elucidate the biological functions of the target proteins of the predicted small molecules and highly correlated genes. The Database for Annotation, Visualization and Integrated Discovery (DAVID) was used for GO analysis of the target proteins of predicted small molecules (Dennis *et al.* 2003). The top three GO terms in the annotation clusters that ranked in the Functional Annotation Clustering function with statistical significance (*P *<0.05) were extracted. The enrichment *P*-values of all extracted GO terms were calculated using DAVID.

### 2.7 Visualization of the PPA network of a group of target proteins of DR-inducing small molecules

To elucidate the association network among target proteins of predicted small molecules, the protein-protein association (PPA) network was constructed from molecular association network data stored in the Search Tool for the Retrieval of Interacting Genes (STRING) Database (Szklarczyk *et al.* 2015) (https://www.string-db.org/). In STRING, protein–protein associations are based on evidence such as experiments, databases, co-expression, neighborhood, gene fusion, and co-occurrence. We used a dataset of 11944806 protein–protein associations of *Mus Musculus* in the STRING database that involved 21291 proteins. We extracted the protein–protein association network involving target proteins of the predicted small molecules and visualized it with Cytoscape (Shannon *et al.* 2003).

The degree of centrality was calculated using Cytoscape to detect nodes with high centrality and depicted by the degree of centrality as the size of the node. The association between proteins was depicted by the “combined score” of STRING as the thickness of the edge. To demonstrate whether the gene expression levels of the target proteins were altered by DR induction, the ratio of expression value of target cells against source cells was depicted by the color of the node using the target cell-specific transcriptome profile. Red indicates that gene expression was upregulated, blue indicates that the gene expression was downregulated, and gray indicates that the gene expression level was unknown.

### 2.8 Evaluation of small molecules that induce DR

To evaluate the proportion of known DR-inducing small molecules or small molecules with similar functions to known DR-inducing ones against the number of all small molecules in the predicted combination, the statistical significance was evaluated by Fisher’s exact probability test.

## 3 Results

### 3.1 Overview of the proposed method for predicting small molecules for DR

**Figure 1.**
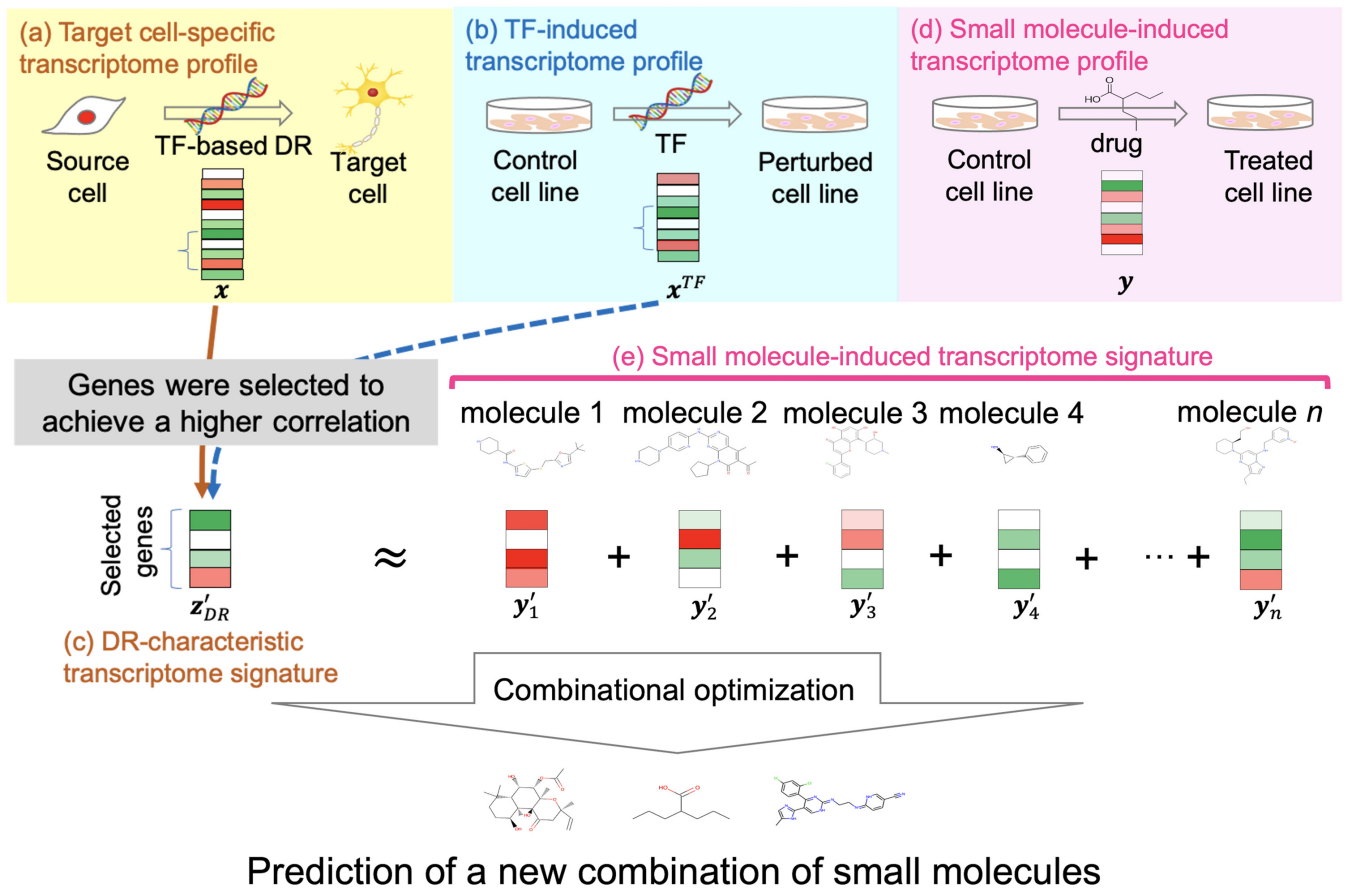
Overview of our proposed method to predict the combination of small molecules that induce DR. Target cell-specific transcriptome profiles for neurons and cardiomyocytes, TF-induced transcriptome profiles, and small molecule-induced transcriptome profiles were constructed. Next, DR-characteristic transcriptome signatures that reflect gene expression patterns of TF-based DR were identified. A new combination of small molecules for DR was predicted by a variant of simulated annealing-based optimization algorithm.

To identify DR-characteristic gene expression patterns, we prepared two types of transcriptome profiles. First, we constructed a set of target cell-specific transcriptome profiles by calculating the expression ratio from the transcriptome profiles of the source cells and the target cells (Fig. 1a). Second, we constructed a TF-induced transcriptome profile, which is the sum of transcriptome profiles in response to the overexpression of genes of DR-inducing TFs (Fig. 1b). Next, we selected correlated genes that have similar expression patterns across the target cell-specific transcriptome profile and TF-induced transcriptome profile, and referred to them as DR-characteristic genes. Finally, we constructed a DR-characteristic transcriptome signature that was defined as the target cell-specific transcriptome profile consisting of only the selected DR-characteristic genes (Fig. 1c).

To identify combinations of small molecules that induce DR, we prepared a set of small molecule-induced transcriptome profiles (Fig. 1d), which included approved drugs. We performed a variant of simulated annealing to explore small molecule combinations on the basis of the correlation between the DR-characteristic transcriptome signature and the sum of small molecule-induced transcriptome signatures (Fig. 1e). In this study, we demonstrated the usefulness of the proposed method through applications of DR of fibroblasts into neurons and cardiomyocytes.

### 3.2 Identification of DR-characteristic transcriptome signatures

To identify DR-characteristic gene expression signatures, we extracted common patterns between target cell-specific transcriptome profiles and TF-induced transcriptome profiles by calculating the correlation coefficients between them. Since not all genes are involved in DR, we removed genes that did not contribute to the common gene expression patterns and selected genes that constituted the DR-characteristic gene expression patterns as DR-characteristic genes. Changing the threshold values of the correlation coefficients little by little, we prepared DR-characteristic genes at different levels. We defined the target-specific transcriptome profile consisting of the selected DR-characteristic genes as the DR-characteristic transcriptome signature. We constructed 11 levels of DR-characteristic transcriptome signatures with correlation coefficient thresholds of 0, 0.5, 0.55, 0.6, 0.65, 0.7, 0.75, 0.8, 0.85, 0.9 and 0.95.

Reportedly, the DR of fibroblasts into neurons is induced by three TFs, Ascl1, Lmx1a, and Nurr1 (Caiazzo *et al.* 2011). Therefore, a DR_neuron_-characteristic transcriptome signature was constructed on the basis of the neuron-specific transcriptome profile and these three TFs-induced transcriptome profiles. For each correlation coefficient, the number of DR_neuron_-characteristic genes was 594, 441, 411, 381, 351, 322, 290, 255, 219, 179, and 135, respectively (Supplementary Fig. S1a and Supplementary Table S1).

Reportedly, the DR of fibroblasts into cardiomyocytes is also induced by three TFs, namely, Tbx3, Mef2c, and Gata4 (Ieda *et al.* 2010). Using the same procedure as per the neurons, the number of DR_cardio_-characteristic genes for each correlation coefficient threshold was 594, 418, 398, 377, 357, 333, 308, 279, 242, 198, and 141, respectively (Supplementary Fig. S1b and Supplementary Table S1).

We first focused on DR_neuron_-characteristic genes of neurons with a correlation coefficient of 0.95 (Supplementary Table S2). To understand the biological functions of these genes, we performed enrichment analysis through gene ontology (GO) and Kyoto Encyclopedia of Genes and Genomes (KEGG) pathway (Kanehisa *et al.* 2009). Multiple GO terms associated with “transcription” were detected (Supplementary Fig. S2a). We then performed the same analyses on DR_cardio_-characteristic genes (Supplementary Fig. S2b). Here, multiple GO terms associated with apoptosis were detected. The term “cell cycle” was enriched in both GO terms and KEGG pathways. Remarkably, “negative regulation of ERK1 and ERK2 cascade,” one of the MAPK signaling cascades associated with cardiac reprogramming, was enriched. These results suggest that DR-characteristic genes of cardiomyocytes are involved in these biological pathways to induce reprogramming of fibroblasts into neurons and cardiomyocytes.

### 3.3 Determination of optimal conditions for predicting small molecules for DR

**Figure 2.**
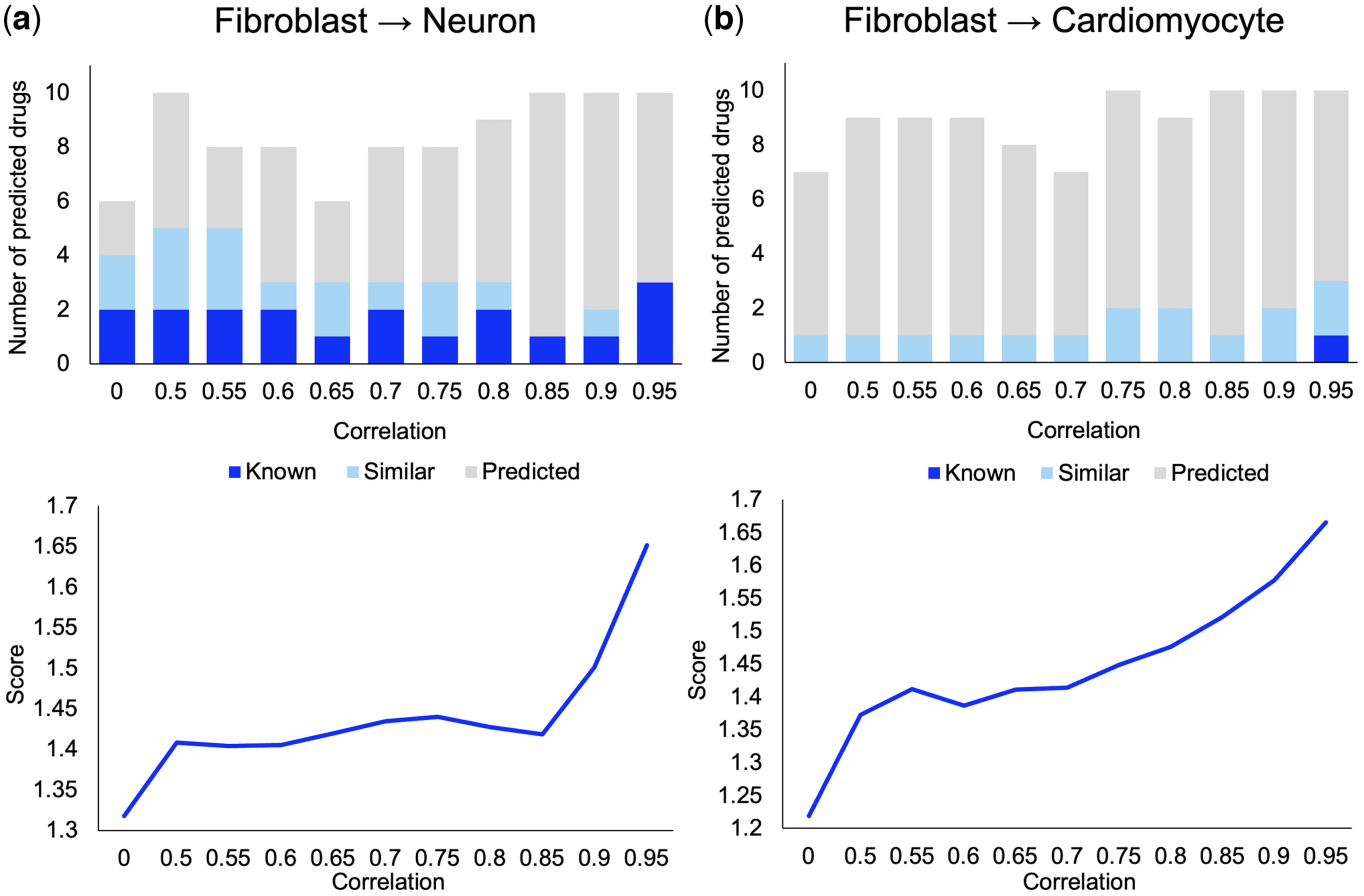
Number of predicted small molecules and the prediction score for the induction of neurons (a) and cardiomyocytes (b). Upper panels show the number of predicted small molecules at each correlation coefficient between target cell-specific and TF-induced transcriptome profiles. Blue bar indicates the number of known DR-inducing small molecules, light blue bar indicates the number of small molecules with similar functions to the known DR-inducing small molecules, and gray bar indicates the number of newly predicted small molecules. The lower panels show the prediction score of the objective function at each correlation coefficient.

We applied our method on DR-characteristic transcriptome signatures at certain correlation coefficients to identify the combination of small molecules that induces DR of neurons and cardiomyocytes. We identified a combination of small molecules that approximate the DR-characteristic transcriptome signature by the sum of the small molecule-induced transcriptome signatures by simulated annealing. Then, we calculated the number of predicted small molecules and prediction scores at each correlation coefficient (Fig. 2).

To determine the small molecules that could drive DR of fibroblast into neurons and cardiomyocytes, we predicted small molecules based on the score of the objective function at each correlation coefficient. Six to ten candidate small molecules were predicted at each correlation coefficient (Fig. 2a and b, Supplementary Table S3a and b). The small molecule list included known DR-inducing small molecules that have been experimentally proven to induce DR and small molecules that have similar biological functions to known ones. The prediction score increased with the correlation coefficient and reached a maximum at a correlation coefficient of 0.95. These results suggest that a correlation coefficient of 0.95 is suitable for predicting the combination of small molecules that convert fibroblasts into neurons and cardiomyocytes.

### 3.4 Identification of the combination of small molecules that induces DR

Using DR-characteristic gene expression signature at the 0.95 correlation coefficient, we predicted the combination of small molecules that induce neurons and cardiomyocytes (Tables 1 and 2). Among predicted combinations of 10 small molecules for DR of neurons, TTNPB (arotinoid acid), romidepsin, and etoposide corresponded to known DR-inducing small molecules (Park *et al.* 2011, Katz *et al.* 2013, Kim *et al.* 2020) (Table 1). We evaluated the ratio of known DR-inducing small molecules among the predicted small molecules by Fisher’s exact test, and confirmed that the known DR-inducing small molecules were significantly present in the predicted small molecules (*P *=0.004503). Additionally, metaraminol and zafirlukast, regulators of neuroactive ligand–receptor interaction, were included among the candidates. These results suggest the potential involvement of DR-inducing small molecules in the regulation of neuronal function.

**Table 1.** List of the small molecules in the predicted combinations for DR of fibroblasts into neurons.

Small molecule name	Efficacy	Function	Pathway
**TTNPB∗**		**Potent RAR agonist**	
**Romidepsin∗**	**Antineoplastic**	**Histone deacetylase inhibitor**	**Cell cycle, Notch signaling pathway, Pathways in cancer**
**Etoposide∗**	**Antineoplastic**	**Topoisomerase II inhibitor**	
Ibrutinib	Antineoplastic	Bruton's tyrosine kinase inhibitor	B cell receptor signaling pathway
Turofexorate isopropyl	Antidyslipidemia	Farnesoid X receptor (FXR) agonist	
Bufexamac	Anti-inflammatory	COX inhibitor	Arachidonic acid metabolism
Metaraminol	Antihypotensive	alpha-Adrenergic receptor agonist	Calcium signaling pathway, Neuroactive ligand-receptor interaction, Adrenergic signaling in cardiomyocytes
Sulfachlorpyridazine	Antibacterial		Folate biosynthesis
Zafirlukast	Antiasthmatic	Leukotriene receptor antagonist	Calcium signaling pathway, Neuroactive ligand-receptor interaction
Ceforanide	Antibacterial	Cell wall biosynthesis inhibitor	Peptidoglycan biosynthesis

Known DR-inducing molecules are highlighted in bold and asterisk. Small molecules with similar functions to known DR-inducing molecules are highlighted in bold.

**Table 2.** List of the small molecules in the predicted combinations for DR of fibroblasts into cardiomyocytes.^a^

Small molecule name	Efficacy	Function	Pathway
**I-BET151∗**		**BET bromodomain inhibitor**	
**Binimetinib**	**Antineoplastic**	**Mitogen-activated extracellular signal-regulated kinase (MEK) inhibitor**	**MAPK signaling pathway, Pathways in cancer, Melanoma**
Pepstatin	Antineoplastic	Pepsin inhibitor	
Calcipotriene	Antipsoriatic	Vitamin D receptor agonist	Endocrine and other factor-regulated calcium reabsorption, Mineral absorption
Gamolenic acid	Analgesic	Anti-inflammatory	
Nifenazone	Analgesic	Antirheumatic	
Adenosine phosphate	Supplement (nutrient)		Neuroactive ligand-receptor interaction
**Filgotinib**	**Anti-inflammatory**	**Janus kinase (JAK) inhibitor**	**JAK-STAT signaling pathway, Th1 and Th2 cell differentiation, Th17 cell differentiation**
Tiratricol	Replenisher (thyroid hormone)		Neuroactive ligand-receptor interaction, Thyroid hormone signaling pathway
Glemanserin	Antianxiety		Neuroactive ligand-receptor interaction, Serotonergic synapse

Known DR-inducing molecules are highlighted in bold and asterisk. Small molecules with similar functions to known DR-inducing molecules are highlighted in bold.

We next looked at the predicted combinations of 10 small molecules for DR of cardiomyocytes (Table 2**)**. I-BET151 is a known DR-inducing small molecule, and binimetinib and filgotinib are small molecules with similar functions to known DR-inducing small molecules (Van Rompaey *et al.* 2013, Woodfield *et al.* 2016, Qin *et al.* 2017). Known DR-inducing small molecules and small molecules with similar functions to known DR-inducing small molecules were significantly present in the predicted small molecules (*P *=0.004503; Fisher’s exact test). Binimetinib and figotinib are known to regulate MAPK and JAK-STAT signaling (Van Rompaey *et al.* 2013, Woodfield *et al.* 2016), which have been reported to be target pathways of DR of cardiomyocytes. Another identified small molecule, gamolenic acid, acts similarly to anti-inflammatory drugs (Kapoor and Huang 2006), and it has been reported to promote DR of cardiomyocytes (Muraoka *et al.* 2019). Altogether, these results suggest that small molecules predicted with our proposed method are involved in multiple biological processes related with DR of cardiomyocytes.

The results with different *T* values (the number of small molecules to be selected) are shown in Supplementary Table S4. When the value of *T* was small (e.g. *T* = 1), no known DR-inducing small molecules were identified.

### 3.5 Molecular mechanisms of the predicted combination of small molecules for DR

To determine the active pathway of the small molecule combination, we examined the molecular mechanisms of the predicted combination of small molecules for DR of neurons and cardiomyocytes. We detected the target proteins of the predicted small molecules in the compound–protein interactome data and evaluated their biological functions and protein–protein associations.

**Figure 3.**
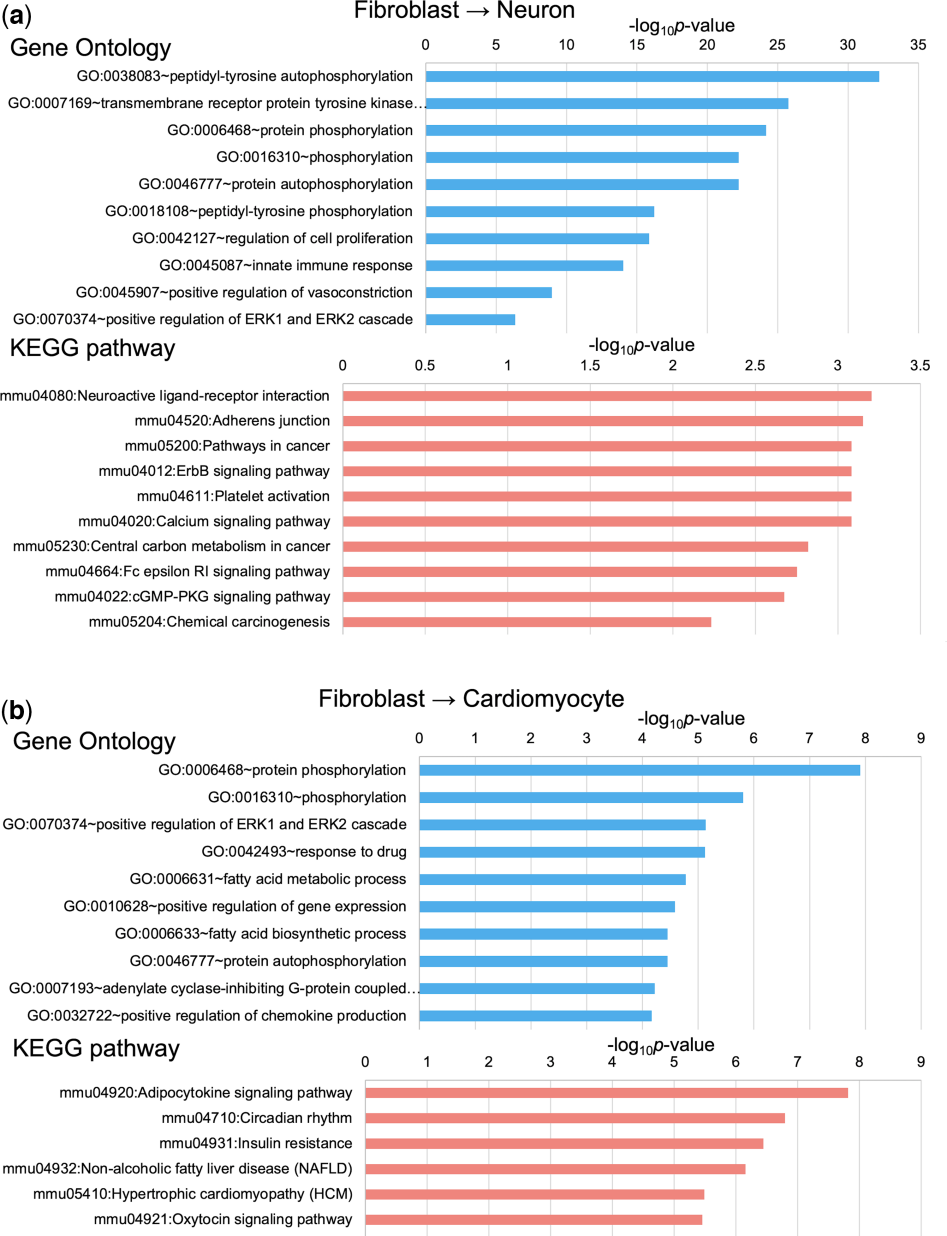
Gene ontology and KEGG pathway enrichment analyses for target proteins of predicted small molecules that induce DR from fibroblasts to neurons and cardiomyocytes. (a) Top 10 GO terms and KEGG pathways of 75 target proteins of predicted small molecules for neurons. (b) Top 10 GO terms and KEGG pathways of 66 target proteins of predicted small molecules for cardiomyocytes.

We detected 75 target proteins for 10 small molecules (Table 1) in the combination predicted for neuronal induction (Supplementary Table S6a). We performed GO and KEGG pathway enrichment analysis on these target proteins (Fig. 3a). Multiple GO terms associated with “protein phosphorylation” were detected, implying that some signaling pathways were activated by the combination of small molecules via protein phosphorylation (Fig. 3a top). ERK1 and ERK2 cascades, common target signaling pathways of cell differentiation and cell reprogramming (Girardi *et al.* 2019, Thomas *et al.* 2020), were detected (Fig 3a top). Moreover, pathways involved in neural activities such as “neuroactive ligand–receptor interaction” and “calcium signaling” were detected in the KEGG pathway (Fig. 3a bottom). These results suggest that the predicted combination of small molecules may induce DR by jointly regulating multiple pathways of neuronal function. Next, we looked at the combination of 10 small molecules (Table 2) predicted for cardiomyocyte induction and obtained 66 target proteins (Supplementary Table S6b). GO analysis of the target proteins (Fig. 3b) detected terms such as “protein phosphorylation” and “positive regulation of ERK1 and ERK2 cascade” (Fig. 3b top), it is similar to neurons-induction linked target proteins. KEGG pathway enrichment analysis detected “hypertrophic cardiomyopathy,” associated with myocardial dysfunction, and “cAMP signaling pathway,” the second messenger that activates intracellular signaling transduction (Fig. 3b bottom). In previous experimental works on small molecule-based DR, Forskolin was often used for induction of several cell types and responsible for activating intracellular signaling by increasing cAMP (Xie *et al.* 2017, Yuan *et al.* 2020). It implies that small molecules in the predicted combination may include candidates that replace forskolin. These results suggest that the predicted combination of small molecules is likely to activate intracellular signaling transduction, resulting in the DR of cardiomyocytes.

**Figure 4.**
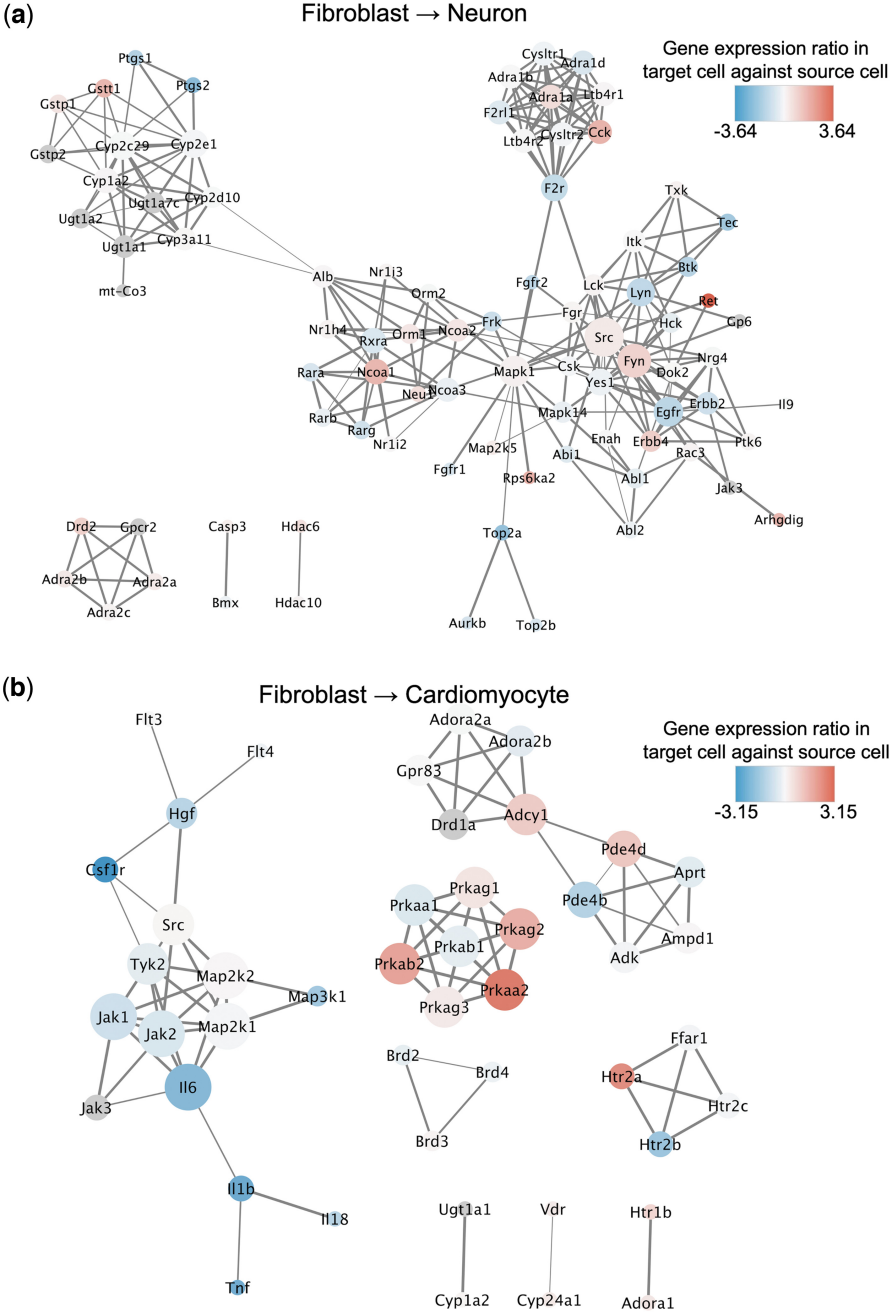
Protein–protein association networks of target proteins of the predicted small molecules that induce DR for neurons (a) and cardiomyocytes (b) from fibroblasts. The size of the nodes indicates the degree of centrality, and the thickness of the edges indicates the protein–protein association scores. The expression ratio is depicted by the color of the node using the target cell-specific transcriptome profile. Red node indicates upregulation of the gene expression level, blue node indicates downregulation of the gene expression level, and gray node indicates that the gene expression level is unknown.

To elucidate the association between target proteins of small molecules in the predicted combination, we examined the target proteins on the protein–protein association (PPA) network (Fig. 4). Mapk1, Src, and Fyn were identified as highly central nodes in the PPA network targeted by the neuron-inducing small molecules (Fig. 4a). Mapk1 is a component protein of the MAPK signaling pathway (Wei and Liu 2002). Src and Fyn are tyrosine–protein kinases of the Src family, and both have been reported to regulate neuronal development through activation of neuronal signaling pathways (Trepanier *et al.* 2012). Therefore, these results suggest that target proteins of predicted small molecules for DR of neurons are involved in neuronal development and functions. We then performed the same analysis on target proteins of small molecules in the combination predicted for cardiomyocytes (Fig. 4b). As a small network of tightly interacted nodes, multiple proteins of AMP-activated protein kinase (AMPK) and the AMPK catalytic subunits (Prkaa1, Prkaa2, Prkab1, Prkab2, Prkag1, Prkag2, and Prkag3) were detected. AMPK are the target proteins of adenosine phosphate, which is activated by increasing cAMP and phosphorylation of PKA (Aslam and Ladilov 2022), and widely regulates cellular functions such as autophagy, metabolism, cytoskeletal signaling autophagy, metabolism, and cytoskeletal signaling (Mihaylova and Shaw 2011). Autophagy and metabolic regulation have been reported as target biological processes that induce DR and cell reprogramming (Xie *et al.* 2017), suggesting that the combination of small molecules targeting AMPK may promote DR. Map2k1 and Map2k2, which are component proteins of the MAPK signaling pathway (Kim and Choi 2010), were detected as highly central target proteins in the network. Additionally, IL6, a cytokine that induces inflammatory responses and crosstalks with MAPK signaling and JAK-STAT signaling (Kagan *et al.* 2017), was detected as a highly central target protein. Gamolenic acid, an anti-inflammatory drug, has been identified as a suppressor of inflammatory response (Muraoka *et al.* 2019) for DR of cardiomyocytes. Gene expression levels of IL6, IL1b, IL18, and Tnf, which are regulators of the inflammatory response (Qin *et al.* 2017), were decreased during the DR of cardiomyocytes. In fact, small molecules in the predicted combination included small molecules that target inflammatory response-regulating proteins. These results suggest that the suppression of inflammatory responses is likely to enhance the DR of cardiomyocytes.

## 4 Discussion and conclusion

In this study, we developed a novel method, DIRECTUER, to replace DR-inducing TFs by DR-inducing small molecules for any direct cell conversions from the integration of TF-inducing transcriptome and small molecule-induced transcriptome data. To the best of our knowledge, this is the first *in silico* method for predicting the combination of small molecules to induce DR based on perturbed transcriptome data. The novelty of the proposed method lies in the extraction of DR-characteristic gene expression patterns, in the development of a variant of simulated annealing to explore the optimal combination of small molecules, and in the applicability to any types of direct cell conversions whose TFs are known. The proposed method enables us to identify the optimal combination of small molecules for DR from a huge number of candidate small molecules and is expected to be useful for promoting research in regenerative medicine.

To induce DR, multiple biological functions must be regulated cooperatively (Qin *et al.* 2017). Previous studies reported that DR is induced by regulating multiple biological functions such as signaling pathways, histone modifications, and other biological processes (e.g. autophagy and metabolic processes) (Qin *et al.* 2017, Xie *et al.* 2017), by altering gene expression in source cells. Additionally, it has been shown that suppression of inflammatory responses enhances cell reprogramming (Muraoka *et al.* 2019). In the case of DR of neurons, we identified a candidate combination of TTNPB, romidepsin, etoposide, and bufexamac (an anti-inflammatory drug). TTNPB regulates signaling pathways (Qin *et al.* 2017, Yuan *et al.* 2020), romidepsin and etoposide regulate histone modifications (Bertino and Otterson 2011, Montecucco *et al.* 2015), and Bufexamac (an anti-inflammatory drug) promotes histone modifications (Bantscheff *et al.* 2011) (Supplementary Fig. S3a). In the case of DR for cardiomyocytes, a candidate combination of I-BET151, binimetinib and filgotinib (two anti-inflammatory drugs) was identified. I-BET151 regulates histone modification (Qin *et al.* 2017, Yuan *et al.* 2020), and binimetinib and filgotinib inhibit MAPK signaling and JAK-STAT signaling, respectively (Van Rompaey *et al.* 2013, Woodfield *et al.* 2016) (Supplementary Fig. S3b). The predicted combinations of small molecules could induce DR by regulating multiple biological processes that are essential for the induction of DR.

Most of the previous studies on DR have focused on experimentally identifying TFs that induce DR for various cell types (Horisawa and Suzuki 2020). However, the risk of tumorigenesis caused by gene insertions is a serious problem, induction of DR by small molecules such as drugs or chemical agents is desired (Federation *et al.* 2014, Qin *et al.* 2017, Xie *et al.* 2017, Yuan *et al.* 2020). DR-inducing small molecules have not been identified yet for most cell types. For example, HNF4α, Foxa3, Gata6 and Cdx2 were identified as DR-inducing TFs for intestinal cells (Miura and Suzuki 2017), but the DR-inducing small molecules have not been established. The proposed method can predict the optimal combination of small molecules on the basis of the list of TFs that are known to induce DR as soon as TF-induced transcriptome profiles are available; thus, the proposed method can be applied to any cell types whenever its DR-inducing TFs have been identified. Hence, the proposed method is expected to be useful for replacing DR-inducing TFs by DR-inducing small molecules, thus reducing the tumorigenic risk due to mutations.

In our proposed method, a combinatorial optimization algorithm was performed to search for combinations of small molecules that induce DR. This method enables the reduction of experimental costs for identifying candidate small molecules from an infinite number of possible small molecules. This was made possible through the restriction of the total number of predictable small molecules by a sub-objective function designed in this study. Nevertheless, there remain parameters that will need to be improved. A related previous method is a regression-based approach (Napolitano *et al.* 2021). There are differences between our proposed method and the previous method. First, the previous method used gene expression data representing each cell type, whereas we used gene expression data during the process of inducing DR. The prediction using our proposed method is performed based on the gene expression profile that reflects the transcriptomic characteristics of DR. Second, the previous method transformed gene expression profiles into pathway-based profiles for prediction, whereas we directly used element values from the gene expression profiles for prediction. A possible research direction would be to take into account optimal experimental conditions such as molecule concentrations and duration time for small molecules.

## Supplementary Material

btae048_Supplementary_Data

## References

[btae048-B1] Aslam M , Ladilov Y. Emerging role of cAMP/AMPK signaling. Cells 2022;11:308. 35053423 10.3390/cells11020308 PMC8774420

[btae048-B2] Bantscheff M , Hopf C, Savitski MM et al Chemoproteomics profiling of HDAC inhibitors reveals selective targeting of HDAC complexes. Nat Biotechnol 2011;29:255–65. 21258344 10.1038/nbt.1759

[btae048-B3] Barrett T , Troup DB, Wilhite SE et al NCBI GEO: mining tens of millions of expression profiles—database and tools update. Nucleic Acids Res 2007;35:D760–5. 17099226 10.1093/nar/gkl887 PMC1669752

[btae048-B4] Bertino EM , Otterson GA. Romidepsin: a novel histone deacetylase inhibitor for cancer. Expert Opin Investig Drugs 2011;20:1151–8. 10.1517/13543784.2011.594437 21699444

[btae048-B5] Cahan P , Li H, Morris SA et al CellNet: network biology applied to stem cell engineering. Cell 2014;158:903–15. 25126793 10.1016/j.cell.2014.07.020 PMC4233680

[btae048-B6] Caiazzo M , Dell'Anno MT, Dvoretskova E et al Direct generation of functional dopaminergic neurons from mouse and human fibroblasts. Nature 2011;476:224–7. 21725324 10.1038/nature10284

[btae048-B7] Černý V. Thermodynamical approach to the traveling salesman problem: an efficient simulation algorithm. J Optim Theory Appl 1985;45:41–51.

[btae048-B8] D’Alessio AC , Fan ZP, Wert KJ et al A systematic approach to identify candidate transcription factors that control cell identity. Stem Cell Rep 2015;5:763–75. 10.1016/j.stemcr.2015.09.016 PMC4649293 26603904

[btae048-B9] Davis RL , Weintraub H, Lassar AB et al Expression of a single transfected cDNA converts fibroblasts to myoblasts. Cell 1987;51:987–1000. 3690668 10.1016/0092-8674(87)90585-x

[btae048-B10] Dennis G Jr , ShermanBT, HosackDA et al DAVID: database for annotation, visualization, and integrated discovery. Genome Biol2003;4:1–11.12734009

[btae048-B11] Federation AJ , Bradner JE, Meissner A et al The use of small molecules in somatic-cell reprogramming. Trends Cell Biol 2014;24:179–87. 24183602 10.1016/j.tcb.2013.09.011 PMC3943685

[btae048-B12] Ferber S , Halkin A, Cohen H et al Pancreatic and duodenal homeobox gene 1 induces expression of insulin genes in liver and ameliorates streptozotocin-induced hyperglycemia. Nat Med 2000;6:568–72. 10802714 10.1038/75050

[btae048-B13] Fu Y , Huang C, Xu X et al Direct reprogramming of mouse fibroblasts into cardiomyocytes with chemical cocktails. Cell Res 2015;25:1013–24. 26292833 10.1038/cr.2015.99 PMC4559819

[btae048-B14] Gaulton A , Bellis LJ, Bento AP et al ChEMBL: a large-scale bioactivity database for drug discovery. Nucleic Acids Res 2012;40:D1100–07. 21948594 10.1093/nar/gkr777 PMC3245175

[btae048-B15] Girardi CS , Rostirolla DC, Lini FJM et al Nuclear RXRα and RXRβ receptors exert distinct and opposite effects on RA-mediated neuroblastoma differentiation. Biochim Biophys Acta Mol Cell Res 2019;1866:317–28. 30529222 10.1016/j.bbamcr.2018.11.014

[btae048-B16] Grath A , Dai G. Direct cell reprogramming for tissue engineering and regenerative medicine. J Biol Eng 2019;13:14–5. 30805026 10.1186/s13036-019-0144-9 PMC6373087

[btae048-B17] Günther S , Kuhn M, Dunkel M et al SuperTarget and matador: resources for exploring drug–target relationships. Nucleic Acids Res 2008;36:D919–22. 17942422 10.1093/nar/gkm862 PMC2238858

[btae048-B18] Guo Z , Zhang L, Wu Z et al In vivo direct reprogramming of reactive glial cells into functional neurons after brain injury and in an alzheimer’s disease model. Cell Stem Cell 2014;14:188–202. 24360883 10.1016/j.stem.2013.12.001 PMC3967760

[btae048-B19] Han X , Yu H, Huang D et al A molecular roadmap for induced multi-lineage trans-differentiation of fibroblasts by chemical combinations. Cell Res 2017;27:386–401. 28128194 10.1038/cr.2017.17 PMC5339836

[btae048-B20] Horisawa K , Suzuki A. Direct cell-fate conversion of somatic cells: toward regenerative medicine and industries. Proc Jpn Acad Ser B Phys Biol Sci 2020;96:131–58. 10.2183/pjab.96.012 PMC7247973 32281550

[btae048-B21] Ieda M , Fu J-D, Delgado-Olguin P et al Direct reprogramming of fibroblasts into functional cardiomyocytes by defined factors. Cell 2010;142:375–86. 20691899 10.1016/j.cell.2010.07.002 PMC2919844

[btae048-B22] Isomi M , Sadahiro T, Fujita R et al Biochemical and biophysical research communications direct reprogramming with Sendai virus vectors repaired infarct hearts at the chronic stage. Biochem Biophys Res Commun 2021;560:87–92. 33984769 10.1016/j.bbrc.2021.04.121

[btae048-B23] Jung S , Appleton E, Ali M et al A computer-guided design tool to increase the efficiency of cellular conversions. Nat Commun 2021;12:1659–12. 33712564 10.1038/s41467-021-21801-4 PMC7954801

[btae048-B24] Kagan P , Sultan M, Tachlytski I et al Both MAPK and STAT3 signal transduction pathways are necessary for IL-6-dependent hepatic stellate cells activation. PLoS One 2017;12:e0176173. 28472150 10.1371/journal.pone.0176173 PMC5417441

[btae048-B25] Kanehisa M , Goto S, Furumichi M et al KEGG for representation and analysis of molecular networks involving diseases and drugs. Nucleic Acids Res 2009;38:D355–60. 19880382 10.1093/nar/gkp896 PMC2808910

[btae048-B26] Kapoor R , Huang Y-S. Gamma linolenic acid: an antiinflammatory omega-6 fatty acid. Curr Pharm Biotechnol 2006;7:531–4. 17168669 10.2174/138920106779116874

[btae048-B27] Katz LS , Geras-Raaka E, Gershengorn MC et al Reprogramming adult human dermal fibroblasts to islet-like cells by epigenetic modification coupled to transcription factor modulation. Stem Cells Dev 2013;22:2551–60. 23627894 10.1089/scd.2013.0134 PMC3760074

[btae048-B28] Kim EK , Choi EJ. Pathological roles of MAPK signaling pathways in human diseases. Biochim Biophys Acta 2010;1802:396–405. 20079433 10.1016/j.bbadis.2009.12.009

[btae048-B29] Kim Y , Jeong J, Choi D et al Small-molecule-mediated reprogramming: a silver lining for regenerative medicine. Exp Mol Med 2020;52:213–26. 32080339 10.1038/s12276-020-0383-3 PMC7062739

[btae048-B30] Kirkpatrick S , Gelatt CD, Vecchi MP et al Optimization by simulated annealing. Science 1983;220:671–80. 17813860 10.1126/science.220.4598.671

[btae048-B31] Knox C , Law V, Jewison T et al DrugBank 3.0: a comprehensive resource for ‘Omics’ research on drugs. Nucleic Acids Res 2011;39:D1035–41. 21059682 10.1093/nar/gkq1126 PMC3013709

[btae048-B32] Li X , Zuo X, Jing J et al Small-molecule-driven direct reprogramming of mouse fibroblasts into functional neurons. Cell Stem Cell 2015;17:195–203. 26253201 10.1016/j.stem.2015.06.003

[btae048-B33] Liu T , Lin Y, Wen X et al BindingDB: a web-accessible database of experimentally determined protein–ligand binding affinities. Nucleic Acids Res 2007;35:D198–201. 17145705 10.1093/nar/gkl999 PMC1751547

[btae048-B34] Ma Y , Xie H, Du X et al In vivo chemical reprogramming of astrocytes into neurons. Cell Discov 2021;7:12. 33649311 10.1038/s41421-021-00243-8 PMC7921425

[btae048-B35] Mihaylova MM , Shaw RJ. The AMPK signalling pathway coordinates cell growth, autophagy and metabolism. Nat Cell Biol 2011;13:1016–23. 21892142 10.1038/ncb2329 PMC3249400

[btae048-B36] Miura S , Suzuki A. Generation of mouse and human Organoid-Forming intestinal progenitor cells by direct lineage reprogramming. Cell Stem Cell 2017;21:456–71.e5. 28943029 10.1016/j.stem.2017.08.020

[btae048-B37] Mohamed TMA , Stone NR, Berry EC et al Chemical enhancement of in vitro and in vivo direct cardiac reprogramming. Circulation 2017;135:978–95. 27834668 10.1161/CIRCULATIONAHA.116.024692 PMC5340593

[btae048-B38] Montecucco A et al Molecular mechanisms of etoposide. EXCLI J 2015;14:95. 26600742 10.17179/excli2015-561 PMC4652635

[btae048-B39] Muraoka N , Nara K, Tamura F et al Role of cyclooxygenase-2-mediated prostaglandin E2-prostaglandin E receptor 4 signaling in cardiac reprogramming. Nat Commun 2019;10:674. 30787297 10.1038/s41467-019-08626-y PMC6382796

[btae048-B40] Nakamura T , Iwata M, Hamano M et al Small compound-based direct cell conversion with combinatorial optimization of pathway regulations. Bioinformatics 2022;38:ii99–105. 36124791 10.1093/bioinformatics/btac475

[btae048-B41] Napolitano F , Rapakoulia T, Annunziata P et al Automatic identification of small molecules that promote cell conversion and reprogramming. Stem Cell Rep 2021;16:1381–90. 10.1016/j.stemcr.2021.03.028 PMC8185468 33891873

[btae048-B42] Park J , Kim C, Tang Y et al Reprogramming of mouse fibroblasts to an intermediate state of differentiation by chemical induction. Cell Reprogram 2011;13:121–31. 21473689 10.1089/cell.2010.0067

[btae048-B43] Qin C , Zhang C, Zhu F et al Therapeutic target database update 2014: a resource for targeted therapeutics. Nucleic Acids Res 2014;42:D1118–23. 24265219 10.1093/nar/gkt1129 PMC3964951

[btae048-B44] Qin H , Zhao A, Fu X et al Small molecules for reprogramming and transdifferentiation. Cell Mol Life Sci 2017;74:3553–75. 28698932 10.1007/s00018-017-2586-x PMC11107793

[btae048-B45] Rackham OJL , Firas J, Fang H et al; FANTOM Consortium A predictive computational framework for direct reprogramming between human cell types. Nat Genet 2016;48:331–5. 26780608 10.1038/ng.3487

[btae048-B46] Roth BL , Lopez E, Patel S et al The multiplicity of serotonin receptors: uselessly diverse molecules or an embarrassment of riches? Neuroscientist 2000;6:252–62.

[btae048-B47] Sadahiro T , Ieda M. In vivo reprogramming as a new approach to cardiac regenerative therapy. Semin Cell Dev Biol 2021;122:21–7. 34210577 10.1016/j.semcdb.2021.06.019

[btae048-B48] Sekiya S , Suzuki A. Direct conversion of mouse fibroblasts to hepatocyte-like cells by defined factors. Nature 2011;475:390–3. 21716291 10.1038/nature10263

[btae048-B49] Shannon P , Markiel A, Ozier O et al Cytoscape: a software environment for integrated models of biomolecular interaction networks. Genome Res 2003;13:2498–504. 14597658 10.1101/gr.1239303 PMC403769

[btae048-B50] Sizykh A , Murtazalieva K, Vyshkvorkina Y et al CFM: a database of experimentally validated protocols for chemical compound-based direct reprogramming and transdifferentiation. F1000Res 2021;10:295.

[btae048-B51] Subramanian A , Narayan R, Corsello SM et al A next generation connectivity map: l 1000 platform and the first 1,000,000 profiles. Cell 2017;171:1437–52.e17. 29195078 10.1016/j.cell.2017.10.049 PMC5990023

[btae048-B52] Szklarczyk D , Franceschini A, Wyder S et al STRING v10: protein–protein interaction networks, integrated over the tree of life. Nucleic Acids Res 2015;43:D447–52. 25352553 10.1093/nar/gku1003 PMC4383874

[btae048-B53] Thomas K et al The mek/erk module is reprogrammed in remodeling adult cardiomyocytes. Int J Mol Sci 2020;21:1–18. 10.3390/ijms21176348 PMC7503571 32882982

[btae048-B54] Trepanier CH , Jackson MF, MacDonald JF et al Regulation of NMDA receptors by the tyrosine kinase Fyn. FEBS J 2012;279:12–9. 21985328 10.1111/j.1742-4658.2011.08391.x

[btae048-B55] Van Rompaey L , Galien R, van der Aar EM et al Preclinical characterization of GLPG0634, a selective inhibitor of JAK1, for the treatment of inflammatory diseases. J Immunol 2013;191:3568–77. 24006460 10.4049/jimmunol.1201348

[btae048-B56] Volarevic V , Markovic BS, Gazdic M et al Ethical and safety issues of stem cell-based therapy. Int J Med Sci 2018;15:36–45. 29333086 10.7150/ijms.21666 PMC5765738

[btae048-B57] Wang H , Yang Y, Liu J et al Direct cell reprogramming: approaches, mechanisms and progress. Nat Rev Mol Cell Biol 2021;22:410–24. 33619373 10.1038/s41580-021-00335-z PMC8161510

[btae048-B58] Wei Z , Liu HT. MAPK signal pathways in the regulation of cell proliferation in mammalian cells. Cell Res 2002;12:9–18. 11942415 10.1038/sj.cr.7290105

[btae048-B59] Woodfield SE , Zhang L, Scorsone KA et al Binimetinib inhibits MEK and is effective against neuroblastoma tumor cells with low NF1 expression. BMC Cancer 2016;16:172–10. 26925841 10.1186/s12885-016-2199-z PMC4772351

[btae048-B60] Xie X , Fu Y, Liu J et al Chemical reprogramming and transdifferentiation. Curr Opin Genet Dev 2017;46:104–13. 28755566 10.1016/j.gde.2017.07.003

[btae048-B61] Xu J , Du Y, Deng H et al Direct lineage reprogramming: strategies, mechanisms, and applications. Cell Stem Cell 2015;16:119–34. 25658369 10.1016/j.stem.2015.01.013

[btae048-B62] Yuan Z-D , Zhu W-N, Liu K-Z et al Small molecule epigenetic modulators in pure chemical cell fate conversion. Stem Cells Int 2020;2020:8890917. 33144865 10.1155/2020/8890917 PMC7596432

