# Supplementary material for: DIRECTEUR: transcriptome-based prediction of small molecules that replace transcription factors for direct cell conversion
Source: Bioinformatics. 2024 Jan 25;40(2):btae048. doi: 10.1093/bioinformatics/btae048 (PMC10868337; doi:10.1093/bioinformatics/btae048)
Supplement: btae048_Supplementary_Data [file btae048_supplementary_data.docx]

Supplementary materials

**DIRECTEUR: Transcriptome-based prediction of small molecules that replace transcription factors for direct cell conversion**

Momoko Hamano^1^, Toru Nakamura^1^, Ryoku Ito^1^, Yuki Shimada^1^, Michio Iwata^1^, Jun-ichi Takeshita^2^, Ryohei Eguchi^1^, Yoshihiro Yamanishi^1,3^

Affiliations:

^1^ Department of Bioscience and Bioinformatics, Faculty of Computer Science and Systems Engineering, Kyushu Institute of Technology, 680-4 Kawazu, Iizuka, Fukuoka 820-8502, Japan, and,

^2^ Research Institute of Science for Safety and Sustainability, National Institute of Advanced Industrial Science and Technology (AIST), 1-1-1, Umezono, Tsukuba, Ibaraki 305-8569, Japan.

^3^ Department of Complex Systems Science, Graduate School of Informatics, Nagoya University, Nagoya 464-8601, Japan

This file includes:

Supplementary Figures and Tables

Supplementary Methods

**Supplementary Figure and Tables**

**Supplementary Table 1** | **The number of removed orthologs at different correlation coefficients.**

| Correlation  coefficient | The number of removed orthologs of neurons | The number of removed orthologs of cardiomyocytes |
| --- | --- | --- |
| 0 | 0 | 0 |
| 0.5 | 153 | 176 |
| 0.55 | 183 | 196 |
| 0.6 | 213 | 217 |
| 0.65 | 243 | 237 |
| 0.7 | 272 | 261 |
| 0.75 | 304 | 286 |
| 0.8 | 339 | 315 |
| 0.85 | 375 | 352 |
| 0.9 | 415 | 396 |
| 0.95 | 459 | 453 |
| 1.0 | 592 | 592 |

**Supplementary Table 2** | **List of highly correlated genes.**

**(a, b)** List of DR-characteristic genes at a correlation coefficient of 0.95 to predict small molecules that induce DR from fibroblasts to neurons **(a)** and cardiomyocytes **(b)**.

| Gene  (a) | | | | |  |
| --- | --- | --- | --- | --- | --- |
| App | Trim13 | Bcl2 | Plekhm1 | Dmtf1 |  |
| Pecr | Mapkapk5 | Skiv2L | Akap8 | Mthfd2 |  |
| Cerk | Trib3 | Iars | Ighmbp2 | Kat6B |  |
| Ube3C | Atf5 | Rad9A | Hmgcr | Kat6A |  |
| Ccnf | Aldh7A1 | Cd320 | Aurkb | Grwd1 |  |
| Ube2L6 | Psmd10 | Scyl3 | Cryz | Ube2A | |
| Arid4B | Copb2 | Ppp1R13B | Rab21 | Lsm5 | |
| Dmp1 | Lrpap1 | Cetn3 | Muc1 | Nfkb2 | |
| Jade2 | Mrpl19 | Hspb1 | Nrip1 | Cnot4 | |
| Ube3B | Stxbp2 | Ptprk | Apbb2 | Adi1 | |
| Cndp2 | Pik3R4 | Ide | Bid | Ppard | |
| Hoxa10 | Slc1A4 | Ikbkap | Ykt6 | Usp14 | |
| Stk10 | Wdr61 | Ado | Smndc1 | Ralb | |
| Nsdhl | Iqgap1 | Gli2 | Polr2K | Pcna | |
| Rgs2 | Htatsf1 | Senp6 | Ptpn1 | Rnmt | |
| Ruvbl1 | Gtf2E2 | Nck2 | Stat1 | Mamld1 |  |
| Capn1 | Tert | Phgdh | Egf | Wrb |  |
| Lgals8 | Dph2 | Nck1 | Nr1H2 | Rpia |  |
| Ccdc92 | Amdhd2 | Dusp3 | Adat1 | Ufm1 |  |
| Hadh | G3Bp1 | Arhgef12 | Rpa1 | Chmp6 | |
| Acot9 | Abl1 | Rbm15 | Xpo7 | Pafah1B1 | |
| Atg3 | Pmaip1 | Ncoa3 | Naga | Hspa4 | |
| Usp7 | Apoe | Axin1 | Mcoln1 | Hprt | |
| Sacm1L | Map2K5 | Itfg1 | Ktn1 | Clpx |  |
| Tpm1 | Decr1 | Npepl1 | Fbxl12 |  |  |
| Cask | Vat1 | Cat | Gstz1 |  | |
| Ddx10 | Cpsf4 | Itgb1Bp1 | Nudt9 |  |  |
| Fbxo11 | Rab4A | Pparg | Eml3 |  |  |

(b)

| Gene | | | | |
| --- | --- | --- | --- | --- |
| Rb1 | Zw10 | Aars | Kit | Stub1 |
| Pecr | Rfx5 | Rad9A | Mcm3 | Cebpz |
| Ube3C | Mapkapk5 | Ddr1 | Pparg | Mamld1 |
| Rrp1 | Slc27A3 | Cep57 | Crk | Lar |
| Clstn1 | Vps28 | Snap25 | Gapdh | Creb1 |
| Plod3 | Abhd4 | Cd40 | Cd44 | Tnip1 |
| Keap1 | Shc1 | Cdkn1B | Pafah1B1 | Slc5A6 |
| Ccp110 | Lpar2 | Ddx42 | Ppard | Msh6 |
| Ube2L6 | Foxo4 | Kif14 | Cab39 | Acly |
| Cblb | Foxo3 | Ikbkap | Heatr1 | Kat6B |
| Eprs | Htatsf1 | Brca1 | Ighmbp2 | Rnps1 |
| Tsen2 | Tert | Ptprf | Bphl | Hyou1 |
| Smc4 | Nup85 | Herpud1 | Lage3 | Gnb5 |
| Pwp1 | Tbxa2R | Gli2 | Aurkb | Iars |
| Rrp8 | Rrs1 | Ngrn | Hdac6 | Lrrc41 |
| Cdc20 | Pmaip1 | Adgre5 | Aurka | Ddb2 |
| Ccnd3 | Wdtc1 | Vps72 | Dnajb2 | Sirt3 |
| Maea | Trap1 | Ctsl | Dnajb1 | Rnf167 |
| Scp2 | Rab4A | Gper1 | Erbb3 | Xpo7 |
| Myc | Hspa4 | Nup62 | Dnajb6 | Tjp1 |
| Fpgs | Ints3 | Timp2 | Nfil3 | Rpia |
| Hadh | Rab27A | Nck2 | Puf60 | Ufm1 |
| Fbxo7 | Ube2A | Casp2 | Ascc3 | Jmjd6 |
| Cog4 | Thap11 | Me2 | Bid | Pin1 |
| Rps6 | Cherp | Rrp12 | Polr2I | Dusp6 |
| Ech1 | Cdk6 | Slc37A4 | Arnt2 | Mok |
| Nosip | Hook2 | Ctsd | Myo10 |  |
| Clpx | Adi1 | Dusp3 | Bad |  |
| Pdia5 | Bcl2 | Mrps2 | Praf2 |  |

**Supplementary Table 3** | **List of predicted small molecules for the induction of neurons and cardiomyocytes in each set of correlated genes.**

| Correlation coefficient | 0 | 0.5 | 0.55 | 0.6 | 0.65 | 0.7 | 0.75 | 0.8 | 0.85 | 0.9 | 0.95 |
| --- | --- | --- | --- | --- | --- | --- | --- | --- | --- | --- | --- |
| Small molecules | Etoposide | Etoposide | Etoposide | Etoposide | Dasatinib | Etoposide | Etoposide | Etoposide | Etoposide | Etoposide | Romidepsin |
|  | Fazarabine | Ethionamide | Panobinostat | Ethionamide | Bufexamac | Dasatinib | Dasatinib | Dasatinib | Norethindrone | Lenvatinib | Etoposide |
|  | Quisinostat | Bufexamac | Irinotecan | Pitavastatin | Irinotecan | Pitavastatin | Bufexamac | Oxolinic | Acifran | Butamben | Ibrutinib |
|  | Dasatinib | Pitavastatin | Ethionamide | Ampicillin | Dalcetrapib | Ethionamide | Triclabendazole | Alitretinoin | Ceforanide | Zafirlukast | Turofexorate |
|  | Tiratricol | Berzosertib | Dalcetrapib | Dasatinib | Ampicillin | Oxolinic | Oxolinic | Ethionamide | Bufexamac | Norethindrone | Bufexamac |
|  | I-BET151 | Oxolinic | Dasatinib | Bufexamac | SB202190 | Bufexamac | Ethionamide | Pitavastatin | Zafirlukast | Heptaminol | Metaraminol |
|  |  | Dasatinib | Bufexamac | Dalcetrapib |  | Erteberel | Pitavastatin | Bufexamac | Ethionamide | Trifluridine | Sulfachlorpyridazine |
|  |  | Baricitinib | SB202190 | SB202190 |  | SB202190 | Tofacitinib | Tolimidone | Tamibarotene | Ethionamide | Zafirlukast |
|  |  | Abexinostat |  |  |  |  |  | Carbon tetrachloride | Trifluridine | Tamibarotene | Ceforanide |
|  |  | SB202190 |  |  |  |  |  |  | Butamben | Etofylline | AGN191183 |

**(a, b)** List of predicted small molecules that induce DR from fibroblasts to neurons **(a)** and cardiomyocytes **(b)** at different correlation coefficients: 0, 0.5, 0.55, 0.6, 0.65, 0.7, 0.75, 0.8, 0.85, 0.9, and 0.95. Column 1 indicates the correlation coefficient value. Columns 2, 3, 4, 5, 6, 7, 8, 9, 10, 11, and 12 to 11 show the predicted small molecules at correlation coefficients of 0, 0.5, 0.55, 0.6, 0.65, 0.7, 0.75, 0.8, 0.85, 0.9, and 0.95, respectively.

(a) Fibroblast→Neuron

| Correlation coefficient | 0 | 0.5 | 0.55 | 0.6 | 0.65 | 0.7 | 0.75 | 0.8 | 0.85 | 0.9 | 0.95 |
| --- | --- | --- | --- | --- | --- | --- | --- | --- | --- | --- | --- |
| Small molecules | Palbociclib | Palbociclib | Palbociclib | Apazone | Daunorubicin | Clebopride | Adenosine | Glemanserin | Carbaryl | Glemanserin | Binimetinib |
|  | Nemonapride | Daunorubicin | Glemanserin | Palbociclib | Etravirine | Etravirine | Cyclosporine | Adenosine | Adenosine | Adenosine | Pepstatin |
|  | Etravirine | Erythromycin | Androstenedione | Daunorubicin | Glemanserin | Glemanserin | Calcipotriene | Apazone | Apazone | Apazone | Calcipotriene |
|  | Plerixafor | Clebopride | Dropropizine | Rivaroxaban | Palbociclib | Nifenazone | Nifenazone | Carbaryl | Pepstatin | Binimetinib | Gamolenic |
|  | Sulfachlorpyridazine | Nelfinavir | Apazone | Nisoldipine | Adenosine | Adenosine | Apazone | Pepstatin | Tiratricol | Nifenazone | Nifenazone |
|  | Daunorubicin | Tiapride | Butalbital | Glemanserin | Androstenedione | Apazone | Carbaryl | Dropropizine | Binimetinib | Pepstatin | Adenosine |
|  | Colchicine | Rivaroxaban | Daunorubicin | Dropropizine | Dropropizine | Binimetinib | Tiratricol | Binimetinib | Nifenazone | Carbaryl | Filgotinib |
|  |  | Apazone | Etravirine | Clebopride | Apazone |  | Glemanserin | Cyclosporine | Dropropizine | Calcipotriene | Tiratricol |
|  |  | Tiratricol | Tiratricol | Tiratricol |  |  | Pepstatin | Nifenazone | Calcipotriene | Filgotinib | Glemanserin |
|  |  |  |  |  |  |  | Binimetinib |  | Glemanserin | Flufenamic | I-BET151 |

(b) Fibroblast→Cardiomyocyte

**Supplementary Table 4** | **List of predicted small molecules for DR in each case of restricting the number of small molecules to be selected.**

**(a, b)** List of predicted small molecules for DR from fibroblasts into neurons **(a)** and cardiomyocytes **(b)** with different numbers of small molecules to be selected (T values). Known DR-inducing small molecules are colored in red.

(a)

(b)

| Small molecule | T=1 | T=2 | T=3 | T=4 | T=5 | T=6 | T=7 | T=8 | T=9 |
| --- | --- | --- | --- | --- | --- | --- | --- | --- | --- |
| 1 | dabrafenib | ceforanide | ceforanide | ceforanide | ceforanide | ceforanide | ceforanide | etoposide | etoposide |
| 2 |  | WAY-362450 | linsitinib | tacalcitol | dabrafenib | TTNPB | etoposide | ceforanide | ceforanide |
| 3 |  |  | WAY-362450 | halofantrine | zafirlukast | ibrutinib | zafirlukast | ibrutinib | TTNPB |
| 4 |  |  |  | tetracaine | tolimidone | etilefrine | ibrutinib | etilefrine | ibrutinib |
| 5 |  |  |  |  | guanfacine | etoposide | TTNPB | TTNPB | zafirlukast |
| 6 |  |  |  |  |  | WAY-362450 | etilefrine | zafirlukast | etilefrine |
| 7 |  |  |  |  |  |  | tolimidone | tolimidone | sulfachlorpyridazine |
| 8 |  |  |  |  |  |  |  | guanfacine | halofantrine |
| 9 |  |  |  |  |  |  |  |  | tetracaine |
| Score | 1.3680629 | 1.4853411 | 1.5313299 | 1.5579379 | 1.5760827 | 1.5978095 | 1.6197145 | 1.6266984 | 1.6372509 |
| P-value | 1.0 | 1.0 | 1.0 | 1.0 | 1.0 | 0.0076890 | 0.0106050 | 0.0139307 | 0.0176461 |

| Small molecule | T=1 | T=2 | T=3 | T=4 | T=5 | T=6 | T=7 | T=8 | T=9 |
| --- | --- | --- | --- | --- | --- | --- | --- | --- | --- |
| 1 | MDL-11939 | MDL-11939 | MDL-11939 | Gamolenic acid | TG-101348 | Gamolenic acid | pepstatin | tiratricol | pepstatin |
| 2 |  | Gamolenic acid | Gamolenic acid | MDL-11939 | pepstatin | tiratricol | tiratricol | Gamolenic acid | Adenosine Phosphate |
| 3 |  |  | TG-101348 | TG-101348 | Gamolenic acid | pepstatin | I-BET-151 | I-BET-151 | nifenazone |
| 4 |  |  |  | hydroquinidine | azelaic-acid | bufexamac | Gamolenic acid | nifenazone | tiratricol |
| 5 |  |  |  |  | tiratricol | I-BET-151 | nifenazone | Adenosine Phosphate | I-BET-151 |
| 6 |  |  |  |  |  | calcipotriol | Adenosine Phosphate | pepstatin | filgotinib |
| 7 |  |  |  |  |  |  | filgotinib | filgotinib | calcipotriol |
| 8 |  |  |  |  |  |  |  | hexylresorcinol | Gamolenic acid |
| 9 |  |  |  |  |  |  |  |  | MDL-11939 |
| Score | 1.4309563 | 1.5254820 | 1.5647611 | 1.586358 | 1.6040026 | 1.6224216 | 1.6341799 | 1.6462239 | 1.652045 |
| P-value | 1.0 | 1.0 | 1.0 | 1.0 | 1.0 | 0.1339938 | 0.15455 | 0.1746482 | 0.1942735 |

**Supplementary Table 5** | **List of predicted small molecules for DR by using differentially expressed genes.**

**(a, b)** List of predicted small molecules for DR from fibroblasts into neurons **(a)** and cardiomyocytes **(b)** by using differentially expressed genes. Known DR-inducing small molecules are colored in red.

(a)

| Small molecules |
| --- |
| Propylene glycol |
| I-BET-151 |
| tetrabenazine |
| clopidogrel |
| hydroxyzine |
| tirofiban |
| SB-202190 |
| orciprenaline |
| dasatinib |

(b)

| Small molecules |
| --- |
| nemonapride |
| clobetasol |
| cinchocaine |
| vindesine |
| thiamphenicol |
| acrivastine |
| benzoic-acid |
| NPI-2358 |
| mofezolac |
| selumetinib |

**Supplementary Table 6** | **List of target proteins of small molecules for the induction of neurons (a) and cardiomyocytes (b).**

**(a, b)** List of target proteins of small molecules at a correlation coefficient of 0.95 that induce DR from fibroblasts to neurons **(a)** and cardiomyocytes **(b)**.

(a)

| Protein (Gene symbol) | | |
| --- | --- | --- |
| FLT3 | AMPD1 | HTR1B |
| FLT4 | TYK2 | PGA5 |
| HTR2B | PRKAB1 | IL6 |
| ADK | APRT | CYP24A1 |
| PRKAG1 | CREB1 | CYP1A2 |
| HTR2C | ADORA2A | IL18 |
| PRKAG2 | ADORA2B | MAP2K1 |
| HTR2A | IL1B | MAP2K2 |
| MPO | ADAM9 | UGT1A1 |
| TNF | MS4A2 | ACSL1 |
| PRKAG3 | PPARA | PDE4D |
| HINT1 | MS4A1 | HGF |
| CASP6 | FBP1 | DRD1 |
| SCN11A | PPARD | BRD4 |
| CTSL | CSF1R | MAP3K1 |
| ADORA3 | PRKAA1 | VDR |
| ADORA1 | PRKAA2 | EIF2AK3 |
| PDE4B | ACSS2 | CTSD |
| FFAR1 | SRC | JAK1 |
| JAK2 | GPR83 | BRD3 |
| CTSE | ADCY1 | BRD2 |
| JAK3 | P2RY2 | PRKAB2 |

(b)

| Protein (Gene symbol) | | |
| --- | --- | --- |
| RET | LTB4R1 | GP6 |
| TOP2A | RARA | BMX |
| ITK | BTK | FAM213A |
| TOP2B | RARB | MAPK14 |
| RARG | CYP2E1 | TEC |
| ORM1 | BLK | PAR2 |
| HDAC10 | SAR | TDP1 |
| FLT3 | SRC | ALB |
| ADRA1D | TXK | CYP1A2 |
| ADRA1B | GSTP1 | FGFR2 |
| ADRA1A | NR1I2 | FRK |
| ORM2 | GSTT1 | MAP2K5 |
| CYSLTR1 | PTGS2 | LYN |
| CYSLTR2 | EGFR | YES1 |
| SLK | NOP9 | NR1H4 |
| AGP2 | HDAC6 | CCK |
| ADORA3 | PTGS1 | PTK6 |
| CASP3 | RXRA | FGR |
| AGP1 | ERBB4 | HCK |
| RAC3 | ERBB2 | AT |
| JAK3 | COX3 | LTB4R2 |
| KCNH2 | ABL1 | CYP2D |
| UGT1A1 | COX2 | LCK |
| RIPK3 | ABL2 | CSK |
| RIPK2 | MAPK1 | DRD2 |
| NCOA3 | COX1 | ADRA2B |
| ADRA2C | FYN | ADRA2A |

**Supplementary Table 7** | **List of predicted small molecules for DR by using the previous method.**

**(a, b)** List of predicted small molecules for DR from fibroblasts into neurons **(a)** and cardiomyocytes **(b)** by using the previous method. Known DR-inducing small molecules are colored in red.

(a)

| Small molecules |
| --- |
| Methotrimeprazine |
| Vinburnine |
| Golvatinib |
| Deferasirox |
| Aminolevulinic |
| I-BET-151 |
| Sufentanil |
| Estriol |
| Methenamine |
| Oxiperomide |

| Small molecules  (b) |
| --- |
| Mequitazine |
| Etravirine |
| Bosutinib |
| Sunitinib |
| Tazobactam |
| Troleandomycin |
| Succinylsulfathiazole |
| Ethaverine |
| Dihydroergotamine |
| Guanadrel |


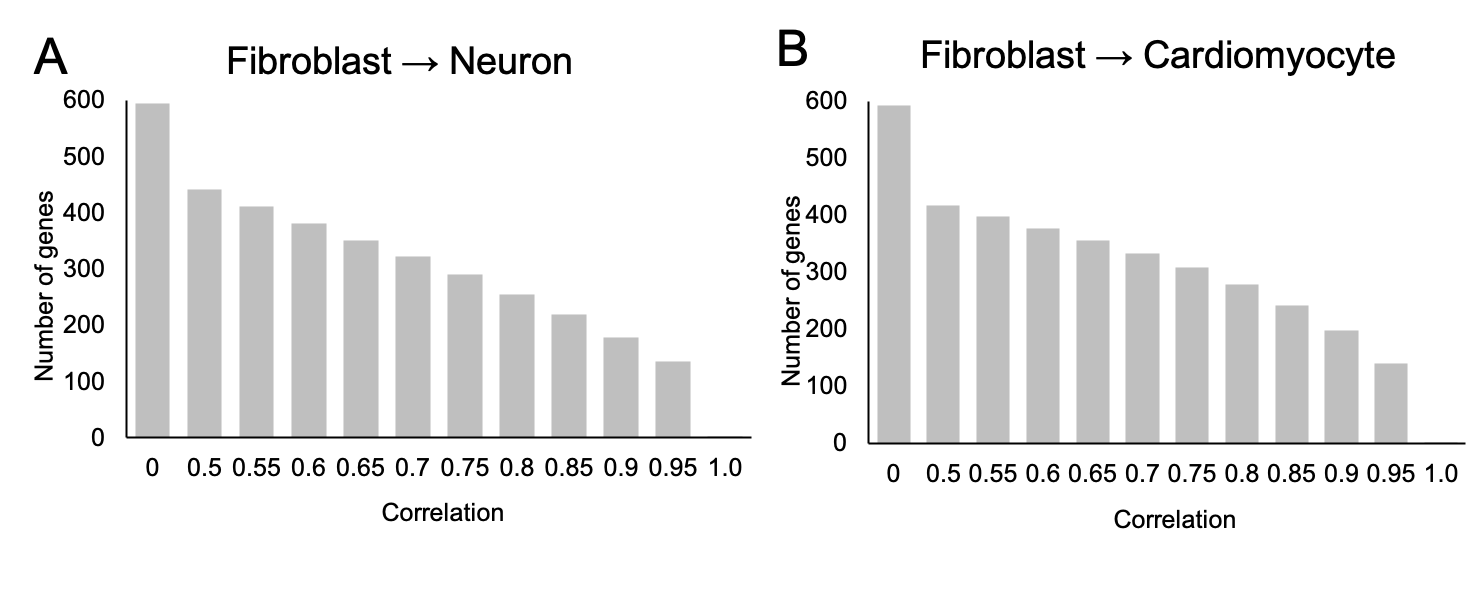


orthologs

orthologs

(b)

(a)

**Supplementary Figure 1** | **The number of corrected orthologs of DR-characteristic orthologs.**

**(a, b)** Number of DR-characteristic orthologs at correlation coefficients of 0, 0.5, 0.55, 0.6, 0.65, 0.7, 0.75, 0.8, 0.85, 0.9, 0.95, and 1.0 used to predict the combination of small molecules that induce DR of fibroblasts into neurons **(a)** and cardiomyocytes **(b)**. The vertical axis indicates the number of orthologs at each correlation coefficient in the bar graphs. (c) The number of removed DR-characteristic orthologs at the correlation coefficients of 0, 0.5, 0.55, 0.6, 0.65, 0.7, 0.75, 0.8, 0.85, 0.9, 0.95, and 1.0.


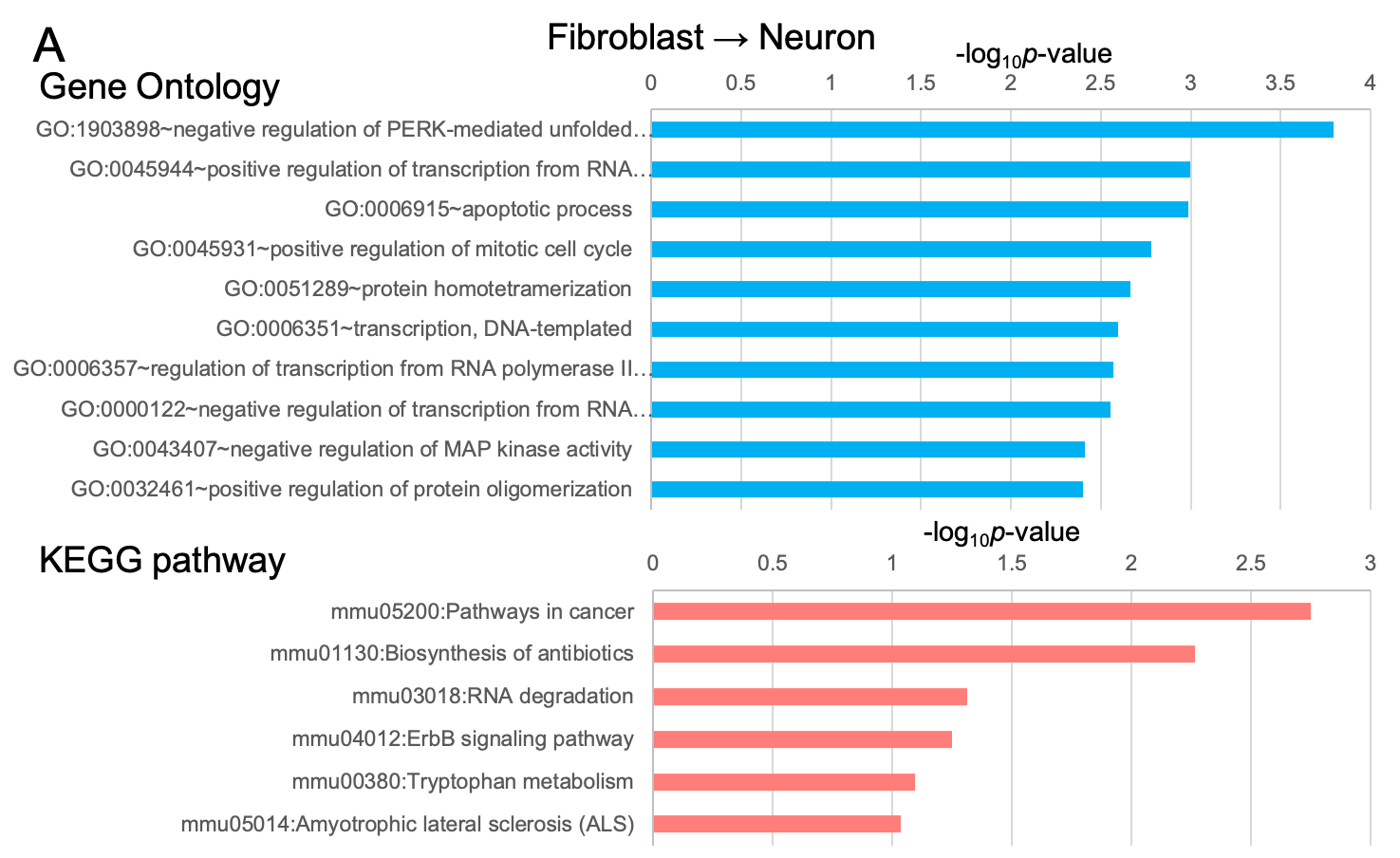

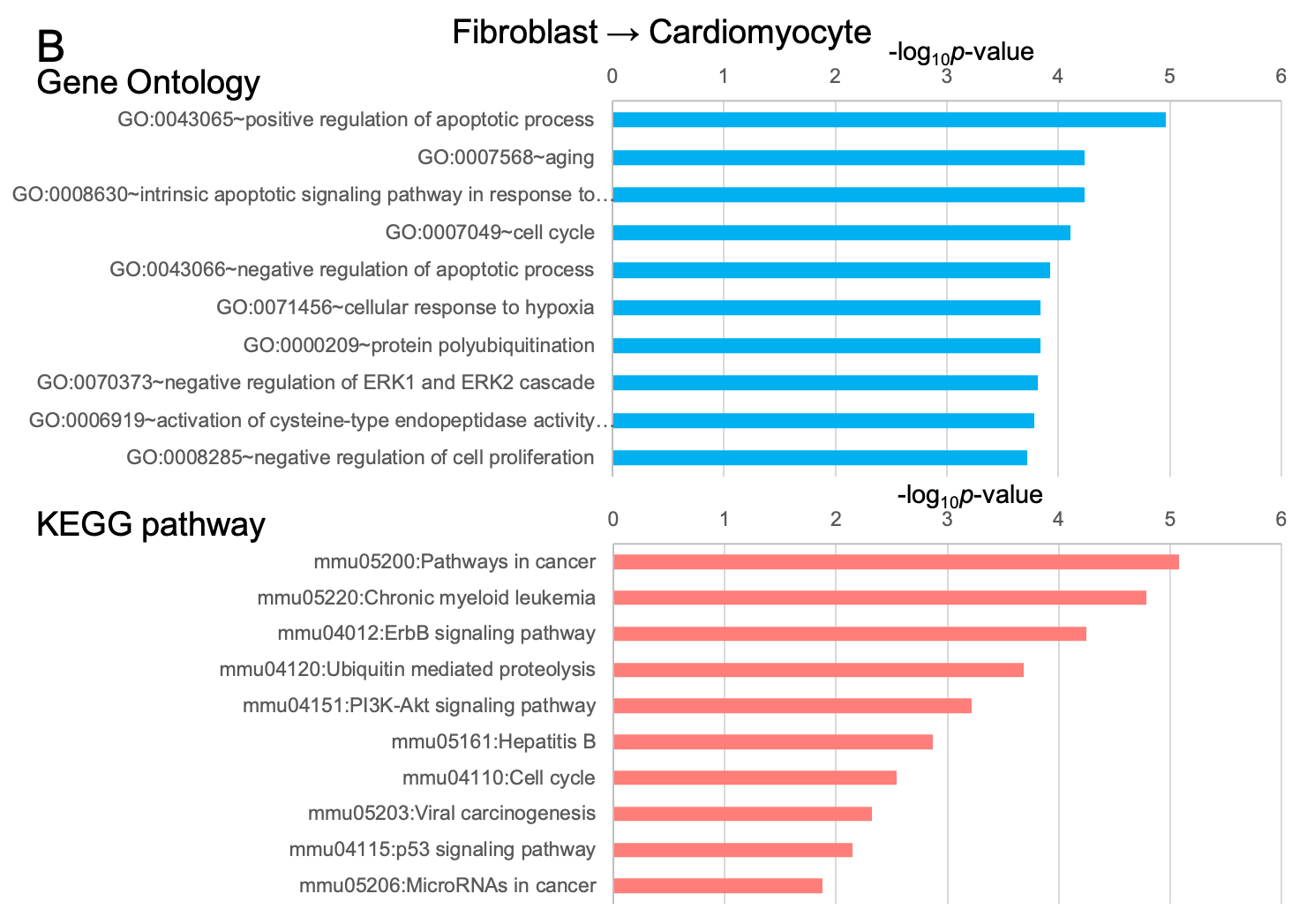


(b)

(a)

**Supplementary Figure 2** | **Gene ontology and KEGG pathway enriched with DR-characteristic genes.**

(a) Top 10 enriched GO terms and KEGG pathways for 135 DR_neuron_-characteristic genes in neurons. **(b)** Top 10 enriched GO terms and KEGG pathways for 141 DR_cardio_-characteristic genes in cardiomyocytes. The upper panel shows the top 10 GO biological process terms, whereas the lower panel shows the top-ranked KEGG pathways. The horizontal axis of each panel represents the –log10 p-value obtained by Fisher’s exact test.

**
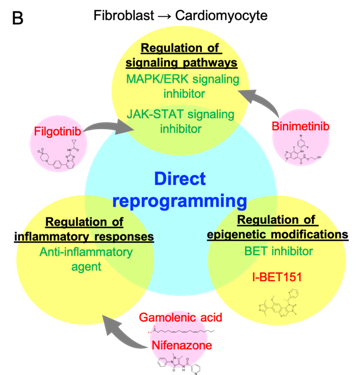

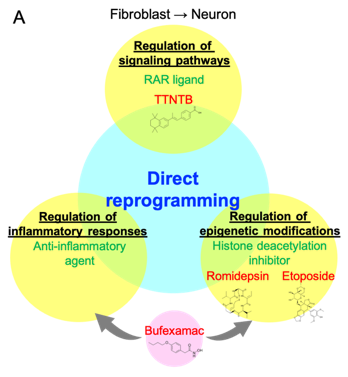
**

(a)

(b)

**Supplementary Figure 3** | **Schematic representation of the molecular mechanisms of predicted small molecules for DR of fibroblasts into neurons (a) and cardiomyocytes (b).**

DR induction involves multiple biological processes such as the regulation of signaling pathways, histone modification, and the inflammatory response (Wang et al., *Cell Stem Cell* (2023)). These schemes show that multiple biological processes are coordinately regulated by predicted small molecules that induce DR. Yellow circles represent the biological processes that induce DR. Biological processes and small molecule names are shown in green and red, respectively. Predicted small molecules in the yellow circle indicate known DR-inducing small molecules. The predicted small molecules in the pink circles function similarly to that of known DR-inducing small molecules. Gray arrows indicate that the predicted small molecules in the pink circles may function in the biological processes in the yellow circle.

**Supplementary Methods**

**Evaluation of small molecules that induce DR**

To evaluate the proportion of known DR-inducing small molecules or small molecules with similar functions to known DR-inducing ones against the number of all small molecules in the predicted combination, the statistical significance was evaluated by Fisher’s exact probability test as follows:

$$p=\frac{\left( a+b \right)!\left( c+d \right)!\left( a+c \right)!\left( b+d \right)!}{n!a!b!c!d!},$$

where *n* is the total number of small molecules, *a* is the number of unknown small molecules among those predicted to induce DR, *b* is the number of known small molecules among those predicted to induce DR, *c* is the number of unknown small molecules among those predicted not to induce DR, and *d* is the number of known compounds among those predicted not to induce DR. The significance level was set at *p* < 0.05.

**The differences between our proposed method and the previous method**

There are three differences between our proposed method and the previous method [Napolitano et al., Stem cell Rep. (2021)]. First, the previous method used gene expression data representing various cell types in FANTOM5, whereas we used gene expression data during the process of inducing DR. The prediction using our proposed method is performed based on the gene expression profile that reflects the transcriptomic characteristics of DR. Second, the previous method transformed gene expression profiles into pathway-based profiles for prediction, whereas we directly used element values from the gene expression profiles for prediction. Third, the strategy for small molecule selection differs between our proposed method and the previous method. The previous method applied a linear regression model, whereas we used a combinatorial optimization algorithm.
